# Proton switching molecular magnetoelectricity

**DOI:** 10.1038/s41467-021-24941-9

**Published:** 2021-07-29

**Authors:** Yong Hu, Scott Broderick, Zipeng Guo, Alpha T. N’Diaye, Jaspal S. Bola, Hans Malissa, Cheng Li, Qiang Zhang, Yulong Huang, Quanxi Jia, Christoph Boehme, Z. Valy Vardeny, Chi Zhou, Shenqiang Ren

**Affiliations:** 1 Department of Mechanical and Aerospace Engineering, University at Buffalo, The State University of New York, Buffalo, NY USA; 2 Department of Materials Design and Innovation, University at Buffalo, The State University of New York, Buffalo, NY USA; 3 Department of Industrial and Systems Engineering, University at Buffalo, The State University of New York, Buffalo, NY USA; 4 Advanced Light Source (ALS), Lawrence Berkeley National Laboratory, Berkeley, CA USA; 5 Department of Physics & Astronomy, University of Utah, Salt Lake City, UT USA; 6 Neutron Scattering Division, Oak Ridge National Laboratory, Oak Ridge, TN USA; 7 Department of Chemistry, University at Buffalo, The State University of New York, Buffalo, NY USA; 8 Research and Education in Energy Environment & Water Institute, University at Buffalo, The State University of New York, Buffalo, NY USA

**Keywords:** Magnetic materials, Ferroelectrics and multiferroics

## Abstract

The convergence of proton conduction and multiferroics is generating a compelling opportunity to achieve strong magnetoelectric coupling and magneto-ionics, offering a versatile platform to realize molecular magnetoelectrics. Here we describe machine learning coupled with additive manufacturing to accelerate the design strategy for hydrogen-bonded multiferroic macromolecules accompanied by strong proton dependence of magnetic properties. The proton switching magnetoelectricity occurs in three-dimensional molecular heterogeneous solids. It consists of a molecular magnet network as proton reservoir to modulate ferroelectric polarization, while molecular ferroelectrics charging proton transfer to reversibly manipulate magnetism. The magnetoelectric coupling induces a reversible 29% magnetization control at ferroelectric phase transition with a broad thermal hysteresis width of 160 K (192 K to 352 K), while a room-temperature reversible magnetic modulation is realized at a low electric field stimulus of 1 kV cm^−1^. The findings of electrostatic proton transfer provide a pathway of proton mediated magnetization control in hierarchical molecular multiferroics.

## Abstract (web summary)

Compared to inorganic materials, the magnetoelectric coupling in macromolecules is still hidden. Here, the authors describe machine learning coupled with additive manufacturing to accelerate the discovery of multiferroic macromolecules with a proton-mediated magnetoelectric coupling effect.

## Introduction

Heterogeneous multiferroics have shown extensive promise for transformative technological applications, making use of strain or Coulomb screening to switch the ferroic orders at interfacial or bulk oxidation states as a means of manipulating magnetoelectric (ME) product properties^1–7^. A transition from multiferroic inorganic heterostructures to macromolecular systems, accompanied by strong moiety dependence of magnetic and ferroelectric properties^8,9^, aqueous processing, and a low coercive field tenability^10,11^, has driven significant research activities. Molecular magnets and ferroelectrics alone (Table S1,S2, molecular magnet, and ferroelectrics) have reached a record high critical temperature (*T*_c_) and outstanding performance compared to inorganic counterparts^10,12–15^. For example, Li_0.7_[Cr(pyrazine)_2_]Cl_0.7_·0.25(tetrahydrofuran) shows a high magnetic ordering *T*_c_ of 515 K, while a giant piezoelectric coefficient d_33_ of around 1540 pC N^−1^ is realized in (TMFM)_0.26_(TMCM)_0.74_CdCl_3_ solid solution (TMFM, trimethylfluoromethyl ammonium; TMCM, trimethylchloromethyl ammonium), comparable to piezoelectric ceramics^12,16^. Compared with single-phase molecular multiferroics with low *T*_c_^17,18^, the high *T*_c_ of molecular magnet and ferroelectrics promise the synthesis of room-temperature molecular multiferroics. Unlike inorganics, molecular materials benefit from their synthetic versatility that results from local coordination and proton chemistries, as well as their large lattice and vacancy network structures that allow precise tailoring and stimuli-dependent modulation of their properties. For example, external species, like ions or protons, could dynamically coordinate with molecular magnets to modulate their magnetism or conductivity^19–21^. However, the effective ME coupling in macromolecules is still hidden or lagging behind their inorganic counterparts. The van-der-Waals-bonded molecular ferroelectrics and magnets become a major challenge for the strain or Coulomb screen mediated ME coupling, due to the weak strictive effect and limited Coulomb screening of molecular insulating building blocks. Despite the coupling challenges, hydrogen-bonded macromolecules, exhibiting rapid proton transfer^20,22–24^, show a promising pathway toward molecular heterogeneous multiferroics with rational design and molecular engineering.

**Fig. 1:**
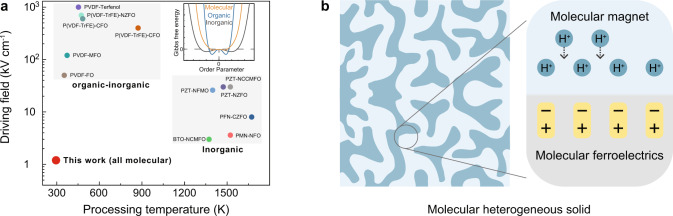
Proton-mediated multiferroic molecular heterogeneous solid. **a** Selected multiferroic composites. Processing temperature is plotted against the driving field (ferroelectric coercivity), as technological important parameters. The low driving field in this work results from the primarily non-covalent bond (i.e., hydrogen bonding) in molecular ferroelectrics which is weaker than the strong ionic/covalent bonds in polymeric and inorganic ferroelectrics. Inset shows the schematic figure for the free energies of molecular, polymeric, and inorganic ferroelectrics^41^. P(VDF-TrFE)-NZFO [Ni_0.5_Zn0_.5_Fe_2_O_4_/poly(vinylidene fluoride-trifluoroethylene)]^42^, PVDF-FO [poly (vinylidene fluoride)-Fe_3_O_4_]^43^, PVDF-Terfenol-D^44^, PVDF-MFO [MnFe_2_O_4_]^45^, P(VDF-TrFE)-CFO [CoFe_2_O_4_]^46^, PZT-NFMO[Pb(Zr_0.52_Ti_0.48_)O_3_–NiFe_1.9_Mn_0.1_O_4_]^47^, PMN-PT-NFO [Pb(Mg_1/3_Nb_2/3_)_0.67_Ti_0.33_O_3_-NiFe_2_O_4_]^48^, PZT-NCCMFO [PZT–NiCo_0.02_Cu_0.02_Mn_0.1_Fe_1.8_O_4_]^49^, PZT-NZFO [PZT-Ni_1−x_ZnxFe_2_O_4_]^50^, BTO-NCMFO [BaTiO_3_-Ni(Co,Mn)Fe_2_O_4_]^51^, PFNO-CZFO [Pb(Fe_0.5_Nb_0.5_)O_3_-Co_0.65_Zn_0.35_Fe_2_O_4_]^52^. **b** Schematic diagram for the proposed proton-mediated ME coupling in molecular heterogeneous solid.

Here we describe machine learning coupled with additive manufacturing to accelerate the discovery of multiferroic heterogeneous macromolecules with a proton-mediated ME coupling effect using a low driving field. A reversible 29% magnetization control at ferroelectric phase transition is observed in such multiferroic heterogeneous macromolecule with a broad thermal hysteresis region of 160 K (between 192 to 352 K). More importantly, a room-temperature reversible magnetic modulation is realized at a low electric field stimulus of 1 kV cm^−1^ (Fig. 1a and inset of Fig. 1a). It should be noted that expensive high temperature and reactive environment growth of inorganic heterogeneous multiferroics is necessary to overcome the perovskite and spinel structure dissimilarity (Fig. 1a); however, such high-temperature processing makes it challenging to intrinsically incorporate protons in the lattice structure. The hydrogen-containing atmospheres and high temperature are therefore indispensable for the proton incorporation and motion in inorganic compounds^25^. The heterogeneous macromolecules described here are grown in aqueous solution at room temperature, with the capability of additive manufacturing, and present an extraordinarily proton-compatible low driving field. In addition, the as-grown molecular ferroelectric building blocks exhibit intrinsic ferroelectric states without poling^22,26^, while the selected molecular magnetic networks show a unique vacancy structure to enable high proton conductivity and proton-tailored magnetism^19,27^. The protons can strongly perturb the electron density of electronegative oxygen in metal-coordinated aqua ligands to effectively tune its magnetism^8,19^. The existing contiguous network of hydrogen-bonded lattice water enables the fast hopping of proton through Grotthuss-type mechanism^28,29^. Based on these unique characteristics of macromolecular magnetic and ferroelectric building blocks, a proton-mediated ME coupling mechanism is proposed. As shown in Fig. 1b, the electric dipole of molecular ferroelectrics can be switched by an external electric field, which can interact with protons in molecular magnets electrostatically. This electrostatic interaction together with the coupling of a proton with magnetism in molecular magnet enables the ME coupling in molecular heterogeneous multiferroics.

## Results

**Fig. 2:**
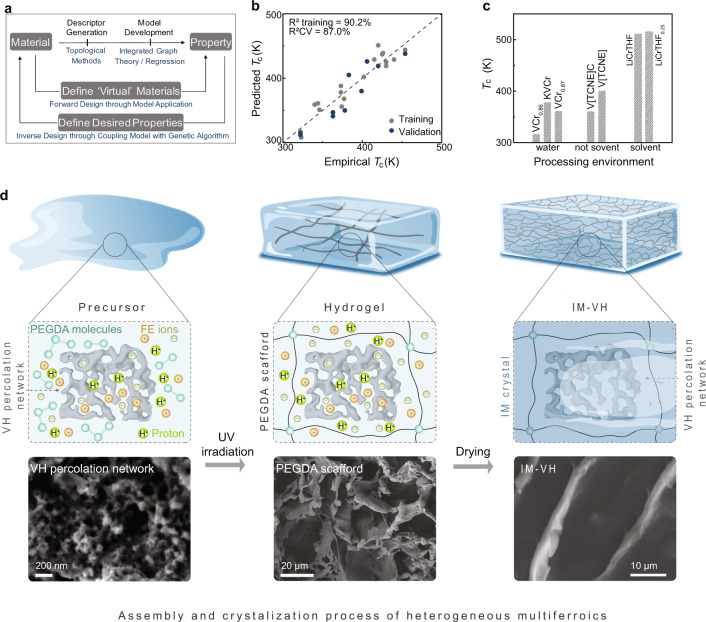
Machine learning design results and solid-solution method for preparing molecular heterogeneous solids. **a** Data-driven computational materials design at the scale of microstructure enabled by supervised machine learning. Training and testing datasets consist of data for each molecular ferroelectric. The machine learning model, typically consisting of a descriptor transformation, a learning algorithm, and an evolutionary inspired optimization aims to discover a statistically correct predictive model from training datasets. **b** Results of the empirical *T*_c_ value (measured data) versus the predicted *T*_c_ value of molecular ferroelectrics from the machine learning models. In all cases, the models are accurate, robust, and can be extended to any molecular system. **c** *T*_c_ and processing environment for selected high-temperature molecular magnets. VCr_0.86_ (V[Cr(CN)_6_]_0.86_*·*2.8H_2_O); KVCr (KV[Cr(CN)_6_]·2H_2_O); VCr_0.87_ (V[Cr(CN)_6_]_0.87_*·*1.6H_2_O); V[TCNE]C (V[TCNE]_x_·yCH_2_Cl_2_); V[TCNE] (V[TCNE]_x_); LiCrTHF (Li_0.7_[Cr(pyz)_2_]Cl_0.7_·(THF)); LiCrTHF_0.25_ (Li_0.7_[Cr(pyz)_2_]Cl_0.7_·0.25(THF)). **d** Schematic diagram for the hydrogel-based method for the multiferroic composites. The hydrogel sample with the desired proton, FE ions, and MA nanoparticles can be obtained by directly printing from the precursor. The crystallization process for the IM-VH is performed based on the printed hydrogel which was dried in a nitrogen atmosphere.

To accelerate the discovery of proton-mediated ME candidates, we applied a machine learning approach for the high-throughput screening of molecular compounds (Fig. 2a). Generally, two basic requirements are considered during the machine learning process: proton-compatible aqueous processability, and high *T*_c_ for room-temperature ME coupling. The results of the analysis are shown in Fig. 2b, c. In the cases of *T*_c_, the regression is set between a nonlinear dimensionality reduction^30,31^ and the quantitative property values, while in the other case a quantitative approach is applied to categorical data to predict the probability that the material can be proton-compatible aqueous processing. A challenge in data-driven design is the requirement of sufficient data. While machine learning often requires huge amounts of data, it should not be an unavailable tool in cases where data is sparser. Instead, it just results in larger uncertainty of modeling. To counter this issue, we have been rigorous in our breakdown of training versus testing data, with a third of the data used for validation. In all cases, the results were within a reasonable spread. Therefore, even with limited data, the results are provided with reasonable confidence and are sufficient for guiding future experimental studies. The selected high *T*_c_ and aqueous processed molecular ferroelectric (Supplementary Fig. 1) and magnetic units through machine learning lead to the accelerated design of proton donors and the candidates for proton-mediated ME heterogeneous solids. We select a model molecular heterogeneous material system to elaborate our design strategy of a proton or ionic control of molecular magnetoelectricity. High T_c_ molecular mixed-valent vanadium hexacyanochromate (VH), a type of Prussian blue analog, is chosen as the magnetic building block due to its unique vacancy network, coordinated and hydrogen bonding guest water molecules to enable proton transfer and magnetism modulation. Water-soluble imidazolium perchlorate (ImClO_4_, IM) is selected as the molecular ferroelectric unit to charge the proton transfer in VH. It should be noted that IM shows as-grown ferroelectricity, essential for the proton storage in VH.

Heterogeneous multiferroic macromolecules are defined by its structure and chemical heterogeneity. Most interesting features in heterogeneous molecules arise at the internal interfaces when the length scale of these inhomogeneities is of the order to match a characteristic charge, spin, and lattice length scale. Achieving such disparate multifunctionalities requires rational design not only of the material itself but also the hierarchical structures at multiple length scales that can respond to external stimuli in real time. The more refined the material building units are the more interfaces increase. The chemical and structurally distinct nature of the molecular magnet and ferroelectric units repel each other during the crystal growth (Supplementary Fig. 2), making it challenging to assemble them into a heterogeneous system by the traditional solution growth method. Thus, a hydrogel-based additive manufacturing strategy is selected for the volumetric design of molecular heterogeneous materials with a large interface at room temperature. The microscale hydrogel cell provides structure confinement to assemble the heterogeneous structure. Figure 2d illustrates the schematic diagram of preparing a model system of molecular IM-VH multiferroics based on hydrogel confinement additive manufacturing strategy. The ionic nature of ferroelectrics IM enables high solubility in water, and the IM solution provides the source of protons due to the weakly nucleophilic perchlorate. Due to the low-density nature (1.6 g cm^−3^) of VH, the molecular magnet can be homogeneously distributed in the precursor and the lifetime of its dispersion can last for more than 1 h without the aggregation settling (Supplementary Fig. 3). The chemical stability of IM and VH enables the maintained characteristic after the ultraviolet (UV) light-induced polymerization process^32^. During the UV light exposure, poly(ethylene glycol) diacrylate (PEGDA) is cross-linked to form the scaffold network with the encapsulated IM ions and VH precursor. The crystal growth of molecular heterogeneous multiferroics is controlled by two processes: the diffusion of ions through the liquid phase to the growth front and the assembly of magnetic components into the molecular ferroelectric crystals.

**Fig. 3:**
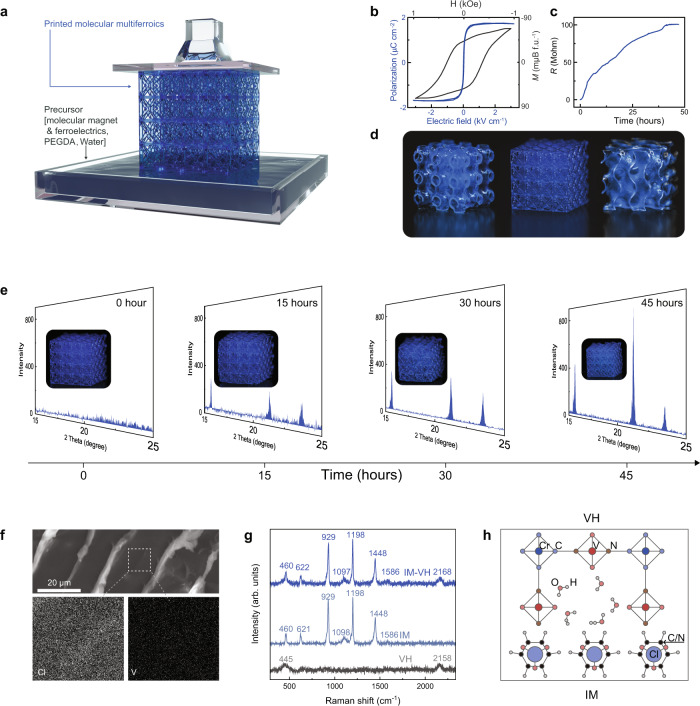
3D printing and crystallization of multiferroic molecular heterogeneous solid. **a** Schematic diagram for manufacturing multiferroic composite (Lattice structure) by the SLA method. The digital projection printing setup (Supplementary Fig. 4) was utilized to project dynamic digital masks on the photocurable polymer composite solution. The UV light used has a wavelength of 385 nm and the control of image projection was achieved through a dynamic micromirror device. **b** Room-temperature polarization vs electric field (P-E) loop for molecular ferroelectric ImClO_4_ at 5 Hz. Room-temperature M-H loop for molecular magnetic MA. **c** Time dependence of resistance measurement during the crystallization process. **d** Optical images for 3D printed IM-VH with different complex geometric structures (Schwarz primitive structure, lattice structure and minimal surface Structures, 25 × 25 × 25 mm^3^). **e** Room-temperature in situ XRD measurement during the crystallization process. Insets show the optical images for the printed sample during the crystallization process. **f** SEM image for IM-VH, bright area indicates the PEGDA scaffold, and the dark area represents IM-VH. EDS mapping for V and Cl elements. **g** Room-temperature Raman spectra for IM-VH, IM, and VH. **h** Schematic diagram for the interface between IM and VH.

An all-molecular assembly strategy with precise spatial control in virtually any geometry is achieved through the three-dimensional (3D) stereolithography, which enables the creation of volumetric architectures consisting of microscale hydrogel cellular confinement features using the programmed automation processes. Figure 3a, Supplementary Fig. 4, and Supplementary Movie 1 illustrate the schematic diagram of printed IM-VH with high dimensional accuracy, structural complexity, and high throughput. Figure 3b shows the magnetic and ferroelectric properties of VH and IM at room temperature. The X-ray magnetic circular dichroism and temperature dependence of magnetization show that VH is ferrimagnetic with a high *T*_c_ of 360 K (Supplementary Fig. 7b), while IM is ferroelectric with *T*_c_ of 373 K^23,33^. Complex geometric heterostructures (Schwarz primitive structure, lattice structure, and minimal surface structures) could be rapidly printed (Fig. 3d).

The as-printed sample is electrically conductive due to the abundance of molecular ions and protons from ferroelectric precursors (Supplementary Fig. 8). In situ resistance measurement (Fig. 3c) shows that the printed sample gradually becomes insulating as the solid crystalizes for the formation of a molecular ferroelectric matrix. Time dependence of X-ray diffraction patterns (XRD) shows that the diffraction intensity gradually increased, revealing the crystallization process. The insets of Fig. 3e are the real-time visualization of the crystallization process. Differential scanning calorimetry (DSC) is performed to study the phase transition behavior of IM-VH. As shown in Supplementary Fig. 9, the distinct exothermic peaks are observed at 220 K for both IM and IM-VH, implying the well crystalized IM in heterogeneous solids. The microstructure of IM-VH is further analyzed. As shown in Fig. 3f, scanning electron microscopy (SEM) and energy dispersive X-Ray spectroscopy (EDS) results show that the skeleton holds the as-printed heterogeneous confinement structure due to the elasticity of the polymer network^34^, while molecular ferroelectric and magnetic phases are uniformly distributed in the scaffold. Supplementary Fig. 10 shows the deformation-dependent flexible IM-VH under loading as a result of the elastic framework.

Raman microscopy measurements (Fig. 3g) study the IM and VH phase in the heterogeneous solids. The peaks at 622, 929, and 1097 cm^−1^ represent the in-plane deformation, symmetric stretching, and asymmetric stretching of the [ClO_4_]^−^ groups, respectively. The peaks for C–H/N–H stretching and C═C/C═N symmetric stretching of imidazolium cations are located at 1149 cm^−1^ and (1448, 1586) cm^−1^, respectively^34^. These peaks show no significant change in the position and the relative intensity, implying the stabilized IM phase. It is noteworthy that the Raman-active mode at 460 cm^−1^ is broad due to its overlap with the out-of-plane deformation vibration of [ClO_4_]^−^ groups and the low-frequency Cr–C stretching and Cr–C bending modes in VH. A broad band at 2110 cm^−1^ can be assigned to the C≡N stretching band, which is sensitive to the bonding mode of the cyanide and to the valance state of the metallic ions coordinated to the C≡N bridge^35^. In addition, the peak of the C≡N stretching mode of VH shifts from 2158 to 2168 cm^−1^, suggesting the interaction between IM and VH. The IM ferroelectric phase controls the proton arrangement in hydrogen-bonded VH through electrostatic force. Absorbance measurements are further performed (Supplementary Fig. 11), in which the band with maxima at 719 nm, assigned to a forbidden transition for [Cr(CN)_6_]^3−^ that becomes allowed when coordinated to V(II)^36^, and show a shift of 8 nm suggesting the interfacial interaction of IM and VH. The interaction also shows an influence on the magnetic property of IM-VH. As shown in Supplementary Fig. 12, the magnetic resonance linewidth of IM-VH is obviously narrower than that of IM from 1 to 17 GHz, suggesting the enhanced magnetic homogeneity in IM-VH^37^.

**Fig. 4:**
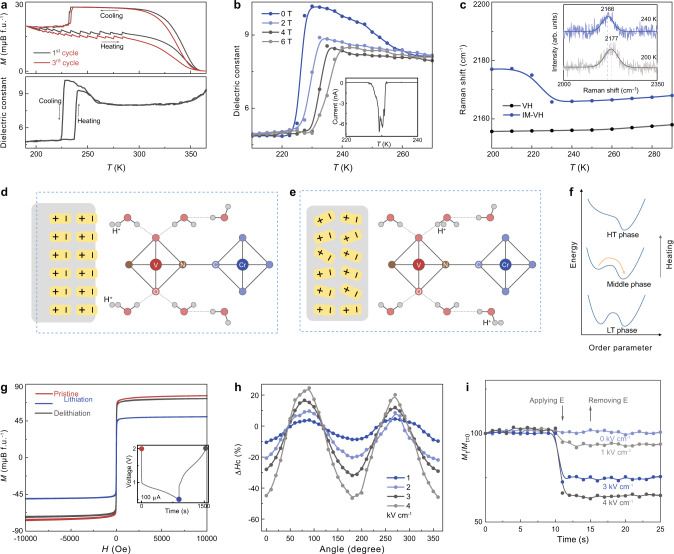
Proton-mediated ME coupling. **a** Temperature dependence of magnetization (*M*) at 10 Oe and dielectric constant (1 kHz) for IM-VH. **b** Temperature dependence of dielectric constant (1 kHz) at different magnetic fields for IM-VH. The inset shows the measured pyroelectric current during the cooling process. **c** Temperature dependence of Raman shift for IM-VH. The inset shows the Raman spectra for IM-VH at 200 and 240 K, the smooth lines are fitting results. **d**–**f** Schematic diagrams for proton-mediated strong coupling between dipole of IM and magnetism of VH. **g** Lithium-ion battery control of magnetism of VH. Inset shows the lithiation and delithiation process. **h** Room-temperature angle dependence of coercivity change for IM-VH solid at different electric fields. 0° means that the applied electric field is parallel to the magnetic field. The x-axis shows the angle value between the applied magnetic field and the magnetic field direction. **i** Room-temperature electric field control of magnetism for IM-VH. The sample was first magnetized at 1 T and then the magnetic field was removed. The electric field was applied along the magnetization direction and the remnant magnetization was monitoring in real time. The switching was found to persist after removing the electric field.

Temperature-dependent magnetization and dielectric measurements (Fig. 4a) show the coupling between the dielectric and magnetic order parameters in the IM-VH heterogeneous solids. This is manifested as a distinct 29% decrease in the magnetization at the first-order structure transition of the molecular ferroelectric phase (232 K). In contrast to the sharp change in magnetization during the cooling process, the magnetization of IM-VH shows a step-like transition with a broad thermal hysteresis width of 160 K (from 192 to 352 K). The transition is repeatable as shown in the first and third cycle temperature-dependent magnetization measurement results. Two possible mechanisms could cause this behavior: strain meditated or proton-mediated nature. The strain effect can be ruled out as the main origin by examining a control experiment on the pressed IM-VH sample (Supplementary Fig. 14), where a weak magnetization change with a subtle thermal hysteresis is observed. Pyroelectric measurement of IM-VH solids (Fig. 4b inset) shows a charge releasing behavior around 232 K, suggesting the role of the ferroelectric material interface to modulate the proton arrangement in VH to achieve proton-mediated magnetism. We note that the cyanide (CN−) groups in VH bridge the transitional metal ions in octahedral crystal fields, splitting their 3d orbitals into *t*_2g_ and *e*_g_ sets, and exhibiting stimuli tunable magnetic transition. One possible mechanism is: the dipoles of IM and protons of VH are interfacially coupled. At the molecular ferroelectric transition temperature, the proton rearrangement leads to the ligand water-metal charge transfer in VH to modulate its magnetism, leading to proton-mediated ME coupling.

Temperature-dependent dielectric measurement further suggests the role of protons on magneto-dielectric coupling in IM-VH. As shown in Fig. 4b, the dielectric transition shifts to a higher temperature with increasing magnetic field while decreasing the dielectric constant. Supplementary Fig. 17a shows that the dielectric constant could be modified by 5% at 230 K under a 4 T magnetic field. Control experiments (Supplementary Figs. 17, 18) confirm that IM or VH phase does not exhibit magnetic field-dependent dielectric behavior. The magnetocapacitance effect obtained in IM-VH further suggests the proton-mediated magnetocapacitance between IM and VH phase. The strain effect from the molecular magnet can be ruled out, as the pressed IM-VH control sample does not exhibit magnetocapacitance.

To provide a mechanistic understanding of magnetoelectricity in IM-VH heterogeneous solids, we further examine temperature-dependent Raman spectra on the C≡N stretching modes (Fig. 4c), which are sensitive to the bonding mode of the cyanide and to the valance state of the metallic ions coordinated to the C≡N bridge. We observe such mode shifts to a high frequency from 200 to 290 K. More importantly, a significantly pronounced shift is observed at the dielectric and magnetic transition region. The Raman spectra indicate that an interfacial interaction induced charge transfer effect has occurred in the metallic ions coordinated C≡N bridge. A proton-mediated ligand-metal charge transfer is proposed here to illustrate such an effect. The interfacial interaction between IM and VH ensures that the surface electric field effect of IM could be effectively applied to the VH phase. According to the literature, such surface electric field of ferroelectrics could reach up to 1 V nm^−1^, coupling the dipole of IM and protons of VH^38^. The VH is surrounded by the IM phase under the ferroelectric field effect (Fig. 4d). During the cooling process, the dipole order is disrupted in IM at 232 K with a first-order phase transition which would induce the proton charge transfer and structure distortion of VH, causing an abrupt magnetic transition. As shown in Fig. 4e, a metastable weak magnetization state can be created. This state is not stable and can be reversed back through thermal-induced transition close to *T*_c_ (Fig. 4f). We also rule out the possibility of the magnetism change induced by the redox reaction in VH as the redox voltage is relatively small. For example, the voltage for Prussian blue, KFe-[Fe(CN)_6_] and K_1.1_Cu_1.15_[Fe(CN)_6_] is about 0.83 and 0.53 V, respectively. Our control experiments also show that the pressed IM and IM-VH do not show electric field-controlled magnetism behavior even 500 V applied. In addition, the lithium-ion control of magnetism study shows that the control of magnetism based on electrochemical redox reaction requires a long switching time (over 1000 s) as shown in Fig. 4g. The short switching time via proton compared with other ionic control could result from its small-sized proton^39,40^.

Proton-mediated coupling between dipole of IM and protons of VH suggests the possibility of electric field control of magnetism at room temperature. As shown in Fig. 4h, the electric field tuning coercivity of IV-VH through proton transfer shows a strong electric field dependence and anisotropic characteristics. A large coercivity change of around −45% is obtained at a low field of 4 kV cm^−1^. The electric field tunable coercivity suggests electric field-controlled magnetism at room temperature (Fig. 4i) in proton-mediated IM-VH heterogeneous solids. The application of an external electric field controls the remnant magnetization of IM-VH and the obtained magnetism control is non-volatile, in which 7, 25, and 36% remnant magnetization decrease is obtained under 1, 3, and 4 kV cm^−1^, respectively. In addition, electric field control of remnant magnetization can be achieved in IM-VH solids under compression up to 19% (Supplementary Fig. 22), showing the feasibility of molecular MEs in flexible electronics. Note that multiferroic heterogeneous PVDF-VH thin film does not show this ME coupling behavior (Supplementary Fig. 23), suggesting the importance of aqua processable proton-mediated heterogeneous solids.

Our study reveals a proton switching ME coupling in a room-temperature molecular heterogeneous multiferroic solid, consisting of chemically dissimilar high *T*_c_ molecular ferroelectric and magnetic moieties. Molecular VH magnet shows a unique lattice structure, such as vacancy network, making it very sensitive to external stimulus and species. The protons and ions that could dynamically incorporate in VH enable its magnetism switching. For molecular ferroelectric IM, its solubility in water and as-grown ferroelectricity make it possible for assembly with a molecular magnet in solution, essential for the development of proton-mediated multiferroics. The multiferroic heterogeneous solids are obtained by a solution-based confinement network enabled by additive manufacturing. With the demonstration of proton-mediated ME coupling, we further demonstrate electric field-controlled magnetism in molecular heterogeneous solids. These findings here could open the pathway to the design of intelligent molecular heterogeneous materials and stimuli-dependent molecular solids, such as molecular magneto-ionics, proton tunable conducting magnets, and voltage switchable molecular spintronics, etc.

## Methods

### Synthesis of imidazolium perchlorate IM and VH

IM was synthesized by allowing the imidazole base dissolved in distilled water to react with perchloric acid. Upon neutralization of the solution, the salt was recrystallized from distilled water. VH was obtained by mixing aqueous solutions (deoxygenated distilled water) of K_3_[Cr(CN)_6_] and VCl_2_. A dark purple solid precipitated immediately and then the solution was kept for 1 day in the glovebox for complete reaction. A dark blue solution was finally formed. The reacted suspension was washed three times with deoxygenated water by centrifugation (6000 rpm, 10 min) to remove the unreacted ions. Traditionally, the obtained compounds were dried under vacuum for 1 day to get the dark blue powder sample. However, the obtained powers are dense and have very large diameters of few micrometers, showing low small specific surface areas. We adopted a method to increase the specific surface areas. Instead of drying under a small vacuum-liquid interface, we prepare a VH thin film by drop-casting and then put the thin film under vacuum (10^−7^ torr) for 1 day to get the dark blue powder sample (Supplementary Fig. 24).

### Hydrogel precursor preparation

Single-phase IM grains were dissolved in purified water to form a saturated solution with a solubility of ~110 g/100 g water. The molecular magnet was added to the saturated IM solution at a different weight ratio (VH/IM = 1–5 wt%). PEGDA (MW 700) was added to the saturated IM solution at a concentration of 20 vol%. Blue-colored dye was added to the precursor of 0.002 wt% in order to have a detailed visualization of the printed part. The precursor was mixed using a sonicator before printing.

### Crystal structure and morphology

The crystal structure was characterized by neutron diffraction, pair distribution function analysis, and Hitachi S4000 Field Emission SEM with IXRF EDS. Neutron scattering experiments were performed on NOMAD at the Spallation Neutron Source (SNS), Oak Ridge National Laboratory.

### Magnetic, dielectric, pyroelectric, and electrical characterization

Magnetic properties were measured by using a vibrating sample magnetometer (VSM) (MicroSense EZ7-380V). X-ray magnetic circular dichroism (XMCD) measurements have been carried out at beamline 6.3.1 at the Advanced Light Source. Sample transfer includes brief (<3 min) to ambient conditions. The measurements were done in total electron yield mode in an alternating magnetic field of 0.5 T to ensure saturation using circularly polarized x-rays from a bend magnet source. No signs of beam damage were observed. Ferromagnetic resonance (FMR) is performed by irradiation of microwave (MW) using a continuous wave (CW) FMR system. FMR system uses a CW broad band MW source (Agilent N5172B-520; 1–19 GHz) which is then connected to a coplanar waveguide (CPW) with sample kept in closed proximity of the waveguide. The MW absorption by the sample is measured via a phase-sensitive detection using a Schottky detector connected to the CPW. Field modulation to the static magnetic field is provided by Helmholtz coils which are needed for locking detection. In pyroelectric measurements, we used capacitor-type samples with a pair of electrodes of silver conducting paint on the parallel faces. The separation between the electrodes was between 2 mm and the area of the cross section was between 9 mm^2^. The pyroelectric effect was measured with Keithley 6512 through a quasi-static method. The cooling rate is 5 K min^−1^. Ferroelectric characterization was performed with a Radiant Ferroelectric Tester Precision LC with a Radiant Precision high voltage interface and a Trek 609B high voltage amplifier. Measurements were performed at room temperature with silver epoxy electrodes. The temperature-dependent dielectric constant at 1 kHz was measured in a Quantum Design Physical Property Measurement System (PPMS) with a Radiant High Voltage Cryogenic Probe and an Agilent 4294 A impedance analyzer with a bias voltage of 0.5 V. Electrical conductivity measurements were performed with a Keithley 2450 SMU.

### Raman microspectroscopy and UV-Vis absorption measurement

A Raman microspectroscopy experiment was performed using a Renishaw inVia Raman microscope (Renishaw, Inc. Hoffman Estates, IL). Spectra were collected using a Renishaw diode laser (488 nm). UV-Vis absorption spectra was collected using an Agilent Cary 7000 UV-Vis-near-IR spectrophotometer.

### Thermal analysis

Thermal transitions were determined using a Perkin Elmer (Shelton, CT) DSC 7 differential scanning calorimeter. Experiments were performed at a heating rate of 10 °C min^−1^. Thermal degradation was measured on a thermogravimetric analyzer (TGA) (SDT Q600, TA Instruments., USA) under N_2_ atmosphere.

### Lyophilization

The lyophilization process was performed with a FreeZone Triad Freeze Dry System (Model 74000). Then the sample was freeze-dried for 24 h and the sample temperature was raised under a high vacuum degree.

### SLA printing process

The computer-aided design model was designed using Creo parametric modeling software (PTC Inc.). The CAD model was then sliced into thin layers and saved as gray-scale images. A custom-programmed slicing software was used to perform the slicing operation, with a layer height of 50 μm. The image resolution is 1280 pixels wide and 800 pixels high, in total containing 1.024 mega-pixels. The building-envelop size is 64.00 mm in width and 40.00 mm in length. The SLA printing process was performed with a custom-built SLA printer, utilizing a bottom-up configuration. The UV light was generated by the light source with a wavelength of 385 nm and the control of image projection was achieved through a dynamic micro-mirror device (DMD, Texas Instruments). The sliced images were transferred from the computer to the DMD chip and the corresponding masks were emitted to the projection plane for printing the corresponding layer. The automation and synchronization of the mask generation and the motion of the build platform were achieved using custom-programmed control software. The motor was connected to the microcontroller and a stepper driver. A custom-made tank with dimensions of 75 mm in width by 60 mm in length by 15 mm in height was fabricated using transparent acrylic sheets (SimbaLux AS3). The acrylic sheets were glued to a glass base, which was coated with polydimethylsiloxane (PDMS, SYLGARD 184, DOW, Inc.). The hydrophobic PDMS base helps to easily separate the cured part from the bottom of the tank.

## Supplementary information

Supplementary Information

Description of Additional Supplementary Files

Supplementary Movie 1

## Data Availability

All relevant data are included within this article and its Supplementary Information files. Any additional information is available from the corresponding author on reasonable request.
