# Supplementary material for: Proton switching molecular magnetoelectricity
Source: Nat Commun. 2021 Jul 29;12:4602. doi: 10.1038/s41467-021-24941-9 (PMC8322162; doi:10.1038/s41467-021-24941-9)
Supplement: Supplementary file 1 — Supplementary Information [file 41467_2021_24941_MOESM1_ESM.pdf]

Supplementary information for

Proton switching molecular magnetoelectricity

Yong Hu[1], Scott Broderick[2], Zipeng Guo[3], Alpha T. N'Diaye[4], Jaspal S. Bola[5], Hans Malissa[5], Cheng Li[6], Qiang Zhang[6], Yulong Huang[1], Quanxi Jia[7], Christoph Boehme[5], Z. Valy Vardeny[5], Chi Zhou[3] & Shenqiang Ren[1,8,9*]

Affiliations:

[1]Department of Mechanical and Aerospace Engineering, University at Buffalo, The State University of New York, Buffalo, NY 14260, USA

[2]Department of Materials Design and Innovation, University at Buffalo, The State University of New York, Buffalo, NY 14260, USA

[3]Department of Industrial and Systems Engineering, University at Buffalo, The State University of New York, Buffalo, NY 14260, USA

[4]Advanced Light Source (ALS), Lawrence Berkeley National Laboratory, Berkeley, CA 94720, USA

[5]Department of Physics & Astronomy, University of Utah, Salt Lake City, Utah 84112, USA

[6]Neutron Scattering Division, Oak Ridge National Laboratory, Oak Ridge, TN 37831, USA

[7]Department of Materials Design and Innovation, University at Buffalo, The State University of New York, Buffalo, New York 14260, USA

[8]Department of Chemistry, University at Buffalo, The State University of New York, Buffalo, New York 14260, USA

[9]Research and Education in Energy Environment & Water Institute, University at Buffalo, The State University of New York, Buffalo, NY 14260, USA

*Correspondence to: shenren@buffalo.edu

Contents

Supplementary Note 1. Machine Learning Procedure 3
Supplementary Note 2. Magnetic and crystal structure of VH 7
Supplementary Table S1. T_c and coercive fields for selected high-temperature molecular magnets. 21
Supplementary Table S2. T_c, and piezoelectric coefficient (d_{33}) (pCN^{-1}) for selected high-temperature molecular ferroelectrics. 21
Supplementary Table S3. 3D printing process parameters 23
Supplementary Table S4. FMR parameters 23
Supplementary References 24

Supplementary Note 1. Machine Learning Procedure

The machine learning approach encompasses multiple stages: the development of a relevant descriptor set, the parameterization of data to avoid over-fitting of the model while maintaining the governing physics, and the development of a high-throughput quantitative structure-property relationship (QSAR). The development of the topological descriptors is based on prior existing approaches which account for both the chemistry/composition, as well as the bonding and stereochemistry[1,2]. This development of descriptors has been used in our prior work for accelerated design of molecular systems[3-5]. For the property values, in the case of Tc the quantitative value is directly used in training the predictive model. Conversely in the case of whether the material can be dissolved in water, we set the values as -1 if it cannot and +1 if it can. In this way, the quantitative approach described here can be applied to categorical data. The output value for the predictions then represents the probability that the material can be proton-compatible aqueous processing (ie. positive values indicate the likelihood to dissolve in water, while negative values indicate the opposite). In both cases, the properties were not input into the data parameterization, so as to allow for prediction of unknown or 'virtual' materials.

Data Parameterization:

The parameterization of the data was done following a non-linear manifold learning approach, and namely the IsoMap algorithm[3,5-8]. This approach generates a graph connecting data points on a high dimensional space to their nearest neighbors, mapped out in the high dimensional space, and then fit to a low dimensional manifold. The objective of the *Isomap* algorithm is to map the distribution of elements in the high dimensional space, represented by the set of data points $\{x_i\} \in R^n$, onto a convex nonlinear manifold M^d of lower dimension $d < n$ and through dimensionality reduction, obtain a two- or three-dimensional embedding of the elements into a weighted graph. The mapping is carried out such that the geodesic distances between the elements in the higher dimensional manifold is preserved when it is mapped onto the lower dimensional graph, so that the edges of the graph are weighted in their length according to the original geodesic distances. The dissimilarity between alloying elements, which themselves form the vertices of this graph, are captured by these distances between them along the edges that connect them to their nearest neighbors. This mapping can be described in set theory as: $x_i \rightarrow y_i \mid y_i \in M^d,\ d<n,\ s.t.$ $\forall(i,j): \left|x_i - x_j\right|_\beta = \left|y_i - y_j\right|_\beta$ where β is a norm, representative of the pairwise geodesic distances d_{ij} between any two elements $'i'$ and $'j'$, which is the curvilinear distance along the manifold in M^d.

In order to construct the initial graph in R^n, we used K nearest-neighbors (KNN), which graphs each point connected by an edge to its $'k'$ nearest neighbors alone $\ni\ d_{ij} = \infty,\ \forall\ \left|i - j\right| > \mathrm{k}$. Among semi-supervised learning KNN has been found to perform well compared to other graphs[9] and was, therefore, employed in the present work. In this work the choice of k was optimized by statistically determining the smallest value that could minimize the residual variance $|\ d_M - d_G\ |$, while providing the maximum number of alternative paths. This ensures that the resulting graph is neither over-connected, leading to loss of pairwise geodesic distances, nor are critical neighbors disconnected. For each data point, we also compute the ratio of the distance to its closest and farthest neighbor. The ratios are then averaged over all data points to calculate a scale-invariant, global parameter, Δ,[10] to estimate the measure of uncertainity introduced by sparsity in high dimensional spaces, given that the data points must have sufficient density on the manifold[11]. Δ can range between zero and one and a small value indicates a healthy variance in pairwise distances.

Development of High Throughput Model:

For property prediction, we used the descriptor set and input it into a graph analysis. This provides a set of parameters, which capture the non-linear relationships in the descriptors. The reason for doing this is to reduce the dimensionality of the input without losing information. That is, we use the graph theory for dimensionality reduction, and thereby can perform a regression with limited risk of over-fitting the model. The reason is that we want to create a parameter space which can be used for all systems - that is, we develop a model based on graph theory parameters, and we need these parameters for all systems of interest.

The regression approach employed was partial least squares (PLS)[11-17]. In PLS the training data is converted to a data matrix with orthogonalized axes, which are based on capturing the maximum amount of information in fewer dimensions. The relationships discovered in the training data can be applied to a test dataset based on a projection of the data onto a high-dimensional hyperplane within the orthogonalized axis-system. With PLS, the properties of the composites can be modeled as a function of the chemical and additive descriptors independent of each other. Typical linear regression models do not properly account for the co-linearity between the descriptors, and as a result the isolated impact of each descriptor on the property cannot be accurately known. However, by projecting the data onto a high-dimensional space defined by axes which are comprised of a linear combination of the composite descriptors and also orthogonalized, the impact of the descriptor on the property can be identified independent of all other descriptors.

PLS finds the maximum variance in the predictor variables (X) and finds the correlation factors between X and the predicted variables (Y) that have maximum variance. In PLS, two linear combinations are generated from the X and Y respectively and the maximum covariance between X and Y is calculated. Consider an X matrix of size $N \times K$ and an $N \times M$ matrix Y. The scores of X, t_a (a=1, 2, …, A=the number of PLS components) are calculated as linear combinations of the original variables with the weights w^*_{ka}. The multidimensional space of X is reduced to the A-dimensional hyper plane. Since the scores are good predictors of Y, the correlation of Y is formed on this hyper plane. The loadings of X (P) represent the orientation of each of the components of the hyper plane. Following this, an accurate and high-throughput equation linking the input.

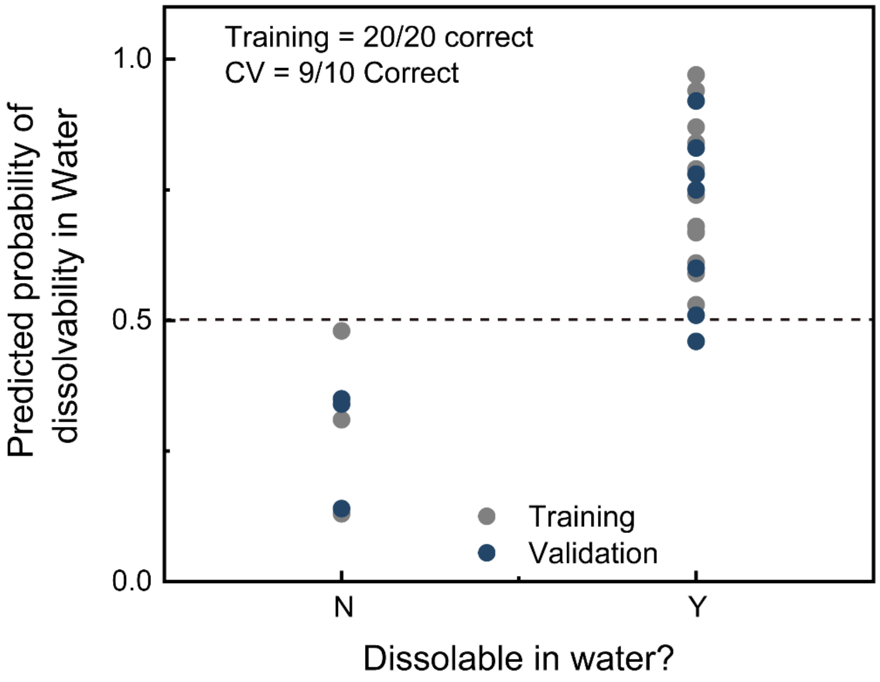

Supplementary Fig. 1. Results for the dissolvable property in water versus the predicted probability of dissolvability in water of molecular ferroelectrics from the machine learning models. Open circles are the training data, while the solid circles are the validation data. In all cases, the models are accurate, and can be extended to any molecular systems.

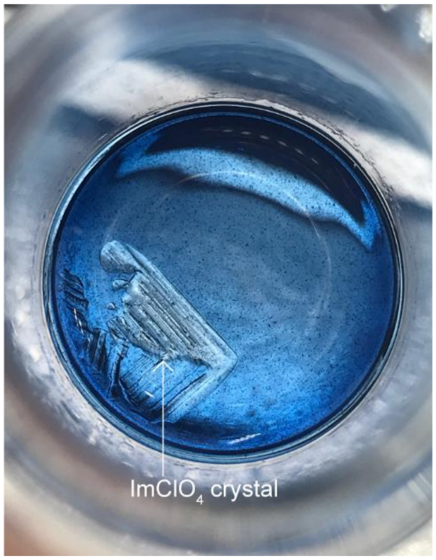

Supplementary Fig. 2. Optical image shows the structure incompatibility of IM and VH. The IM crystal are growth from the solution of IM and VH and VH is expelled from the IM crystal.

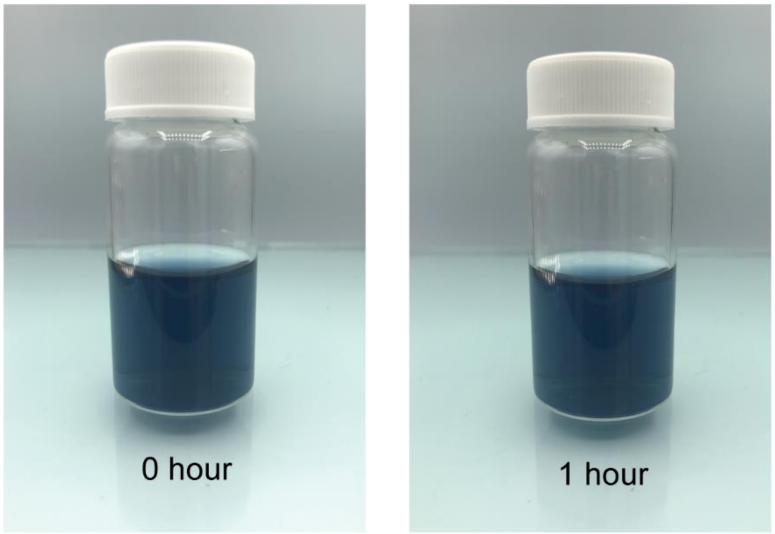

Supplementary Fig. 3. Optical image for precursor at 0 hour and after 1 hour.

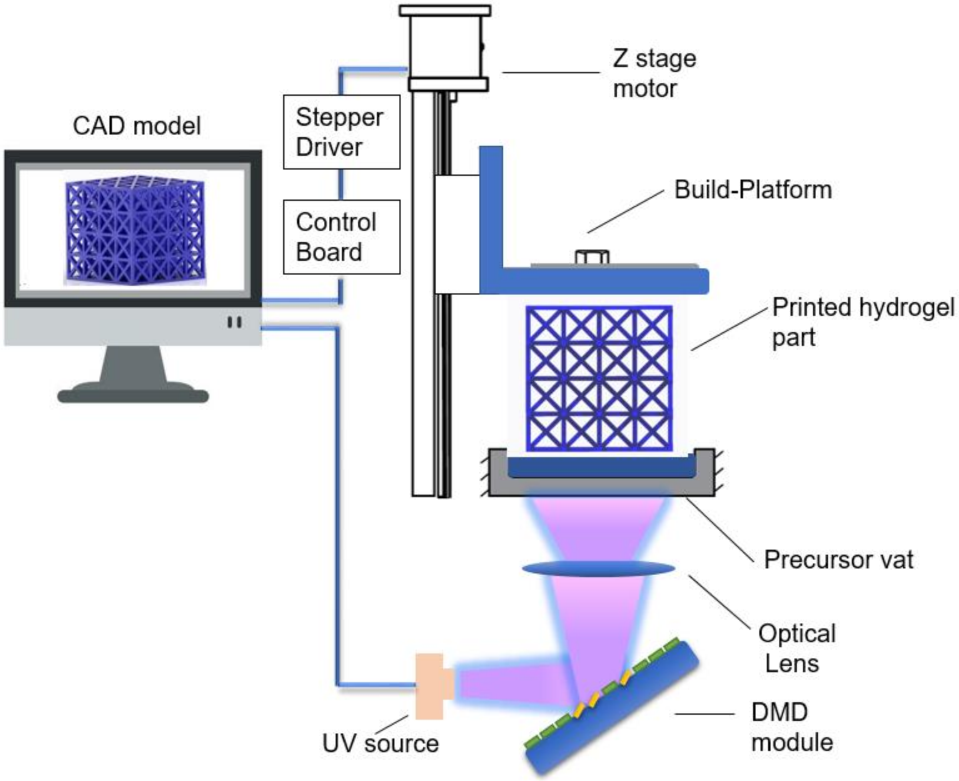

Supplementary Fig. 4. Schematic figure for 3D printing process.

Supplementary Note 2. Magnetic and crystal structure of VH

To determine the composition, we performed the TGA, SEM and EDS measurements. TGA was performed under N_2 atmosphere from 300 K to 470 K[18-20]. The obtained composition of sample is $V[Cr(CN)_6]_{0.87}\cdot 1.6H_2O$. Room-temperature neutron scatting experiment show that the nearest bond length for C-N, C-Cr and N-V bonds are 1.145 Å, 2.035 Å and 2.125 Å, respectively.

Temperature dependence of magnetic susceptibility confirms $T_c \approx 360$ K of VH. FMR measurement give an obvious resonance signal. The *XMCD* spectroscopy provides the understanding of the magnetic exchange interaction in VH. The isotropic spectra at the Cr edge and at the V edge in VH display the main features expected in the $L_{2,3}$ edge of a transition metal ion in nearly octahedral surroundings: at the V's $L_{2,3}$ edge, the dichroic signal is first positive and then negative, whereas at the Cr's $L_{2,3}$ edge, it behaves oppositely. The inversion of the dichroic signal in V compared to Cr indicate the existence of antiferromagnetic coupling between V and Cr atoms.

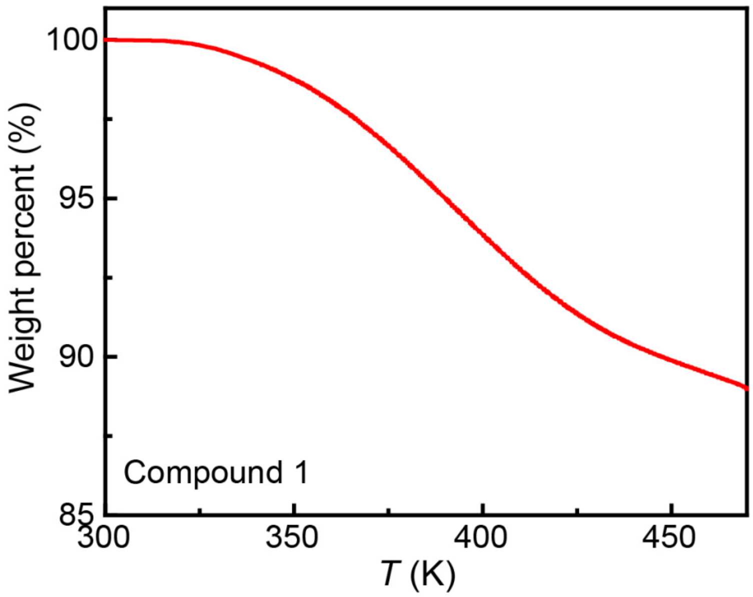

Supplementary Fig. 5. TGA for VH from 300K to 470K.

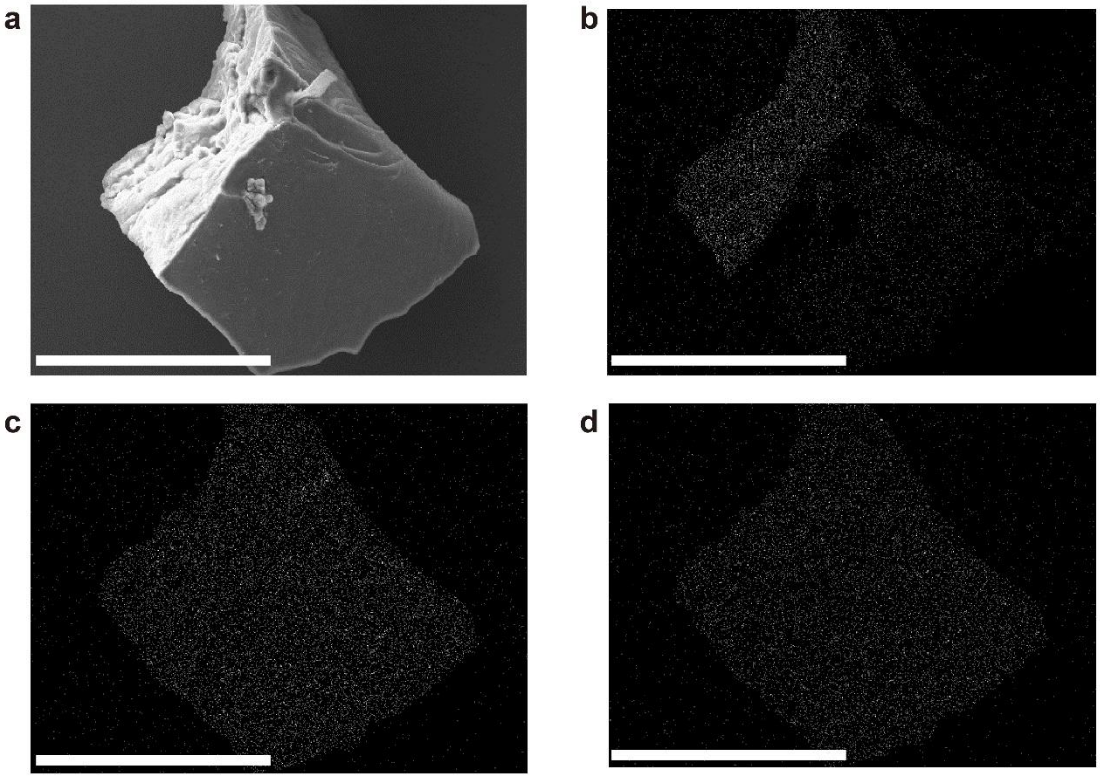

Supplementary Fig. 6. Morphology and elemental mapping of compound 1. **a**, SEM, EDS mapping of **b**, C element, **c**, V element and **d**, Cr element. The scale bar is 100 μm.

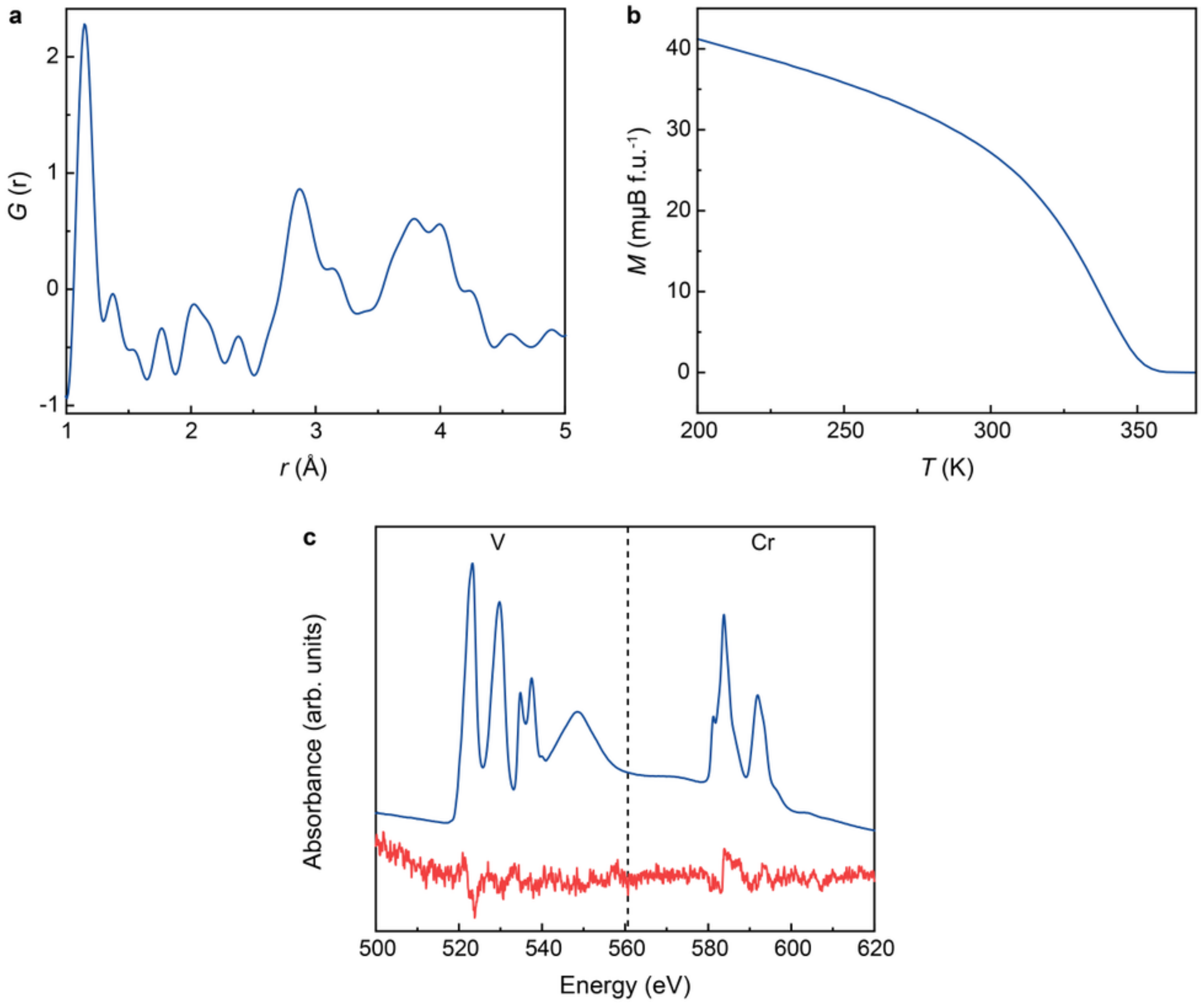

Supplementary Fig. 7. Crystal and magnetic property of VH. **a**, Pair distribution functions, G(r), for VH. **b**, Magnetization (M) in milli-Bohr magnetons per formula unit (m μB f.u.$^{-1}$) versus temperature curves measured at 10 Oe. **c**, Chromium and vanadium $L_{2,3}$ edge in compound 1: dichroic absorption spectra.

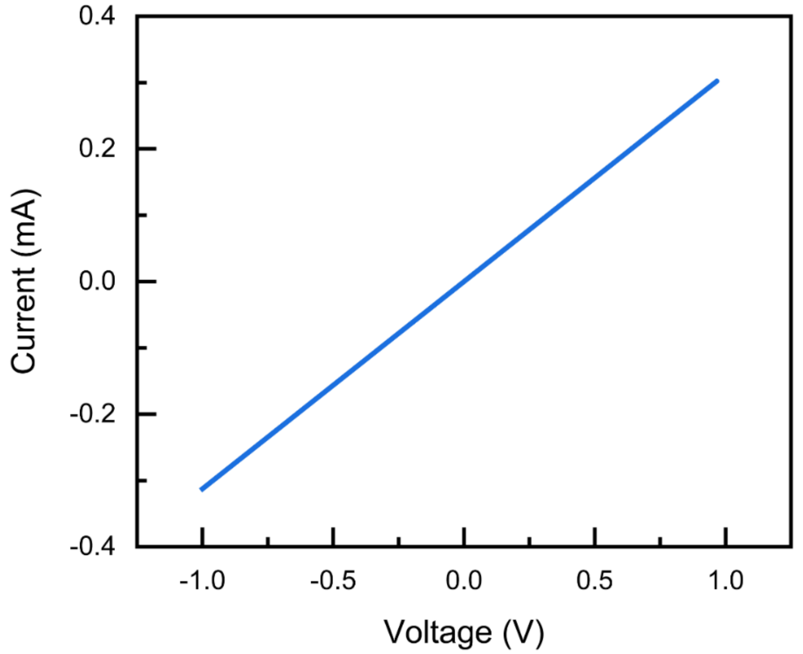

Supplementary Fig. 8. Current-voltage (I-V) curve for printed IM-VH hydrogel before drying.

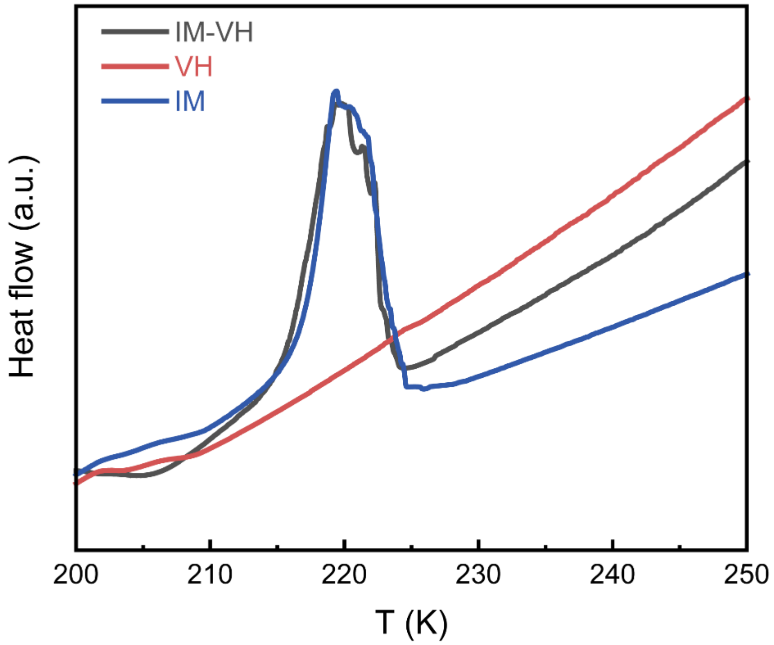

Supplementary Fig. 9. DSC curves for IM. VH and IM-VH.

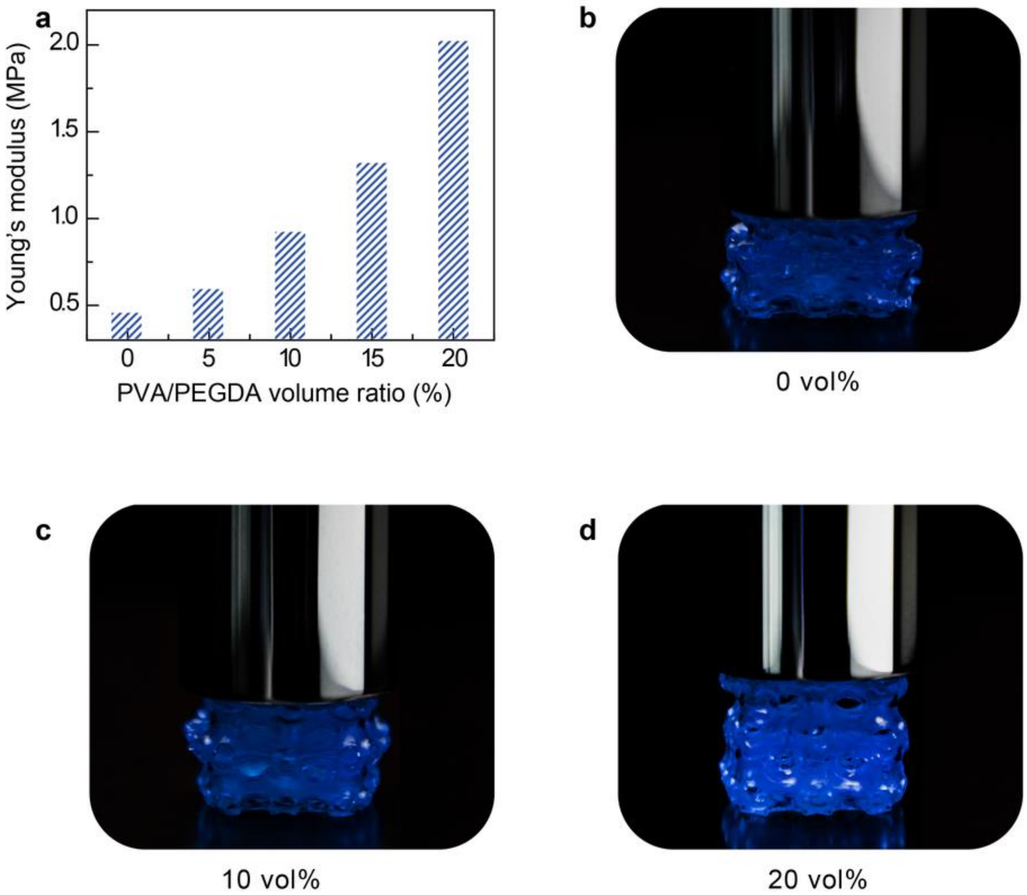

Supplementary Fig. 10. a, Young's modulus for printed IM-VH with different PVA/PEGDA volume ratio. Optical images show the deformation under 100g loading for IM-VH with different PVA/PEGDA volume ratio. **b**, 0 vol%. **c**, 10 vol%. **d**, 20 vol%.

Polyvinyl alcohols (PVA) is a synthetic biodegradable thermoplastic polymer which is nontoxic and has application in different fields such as resins, medical, building industry, packaging materials etc. The better interfacial adhesion between the PVA and PEGDA, PVA composites were mechanically stronger and tougher than the PVA film which results in wide application of PVA for composite material. Young's Modulus of printed PBA-IM with different PVA concentration is measured. It is obvious that the Young's modulus increased from 0.4 MPa to 2.0 MPa as the PVA/PEGDA volume ratio increased from 0 % to 20 %.

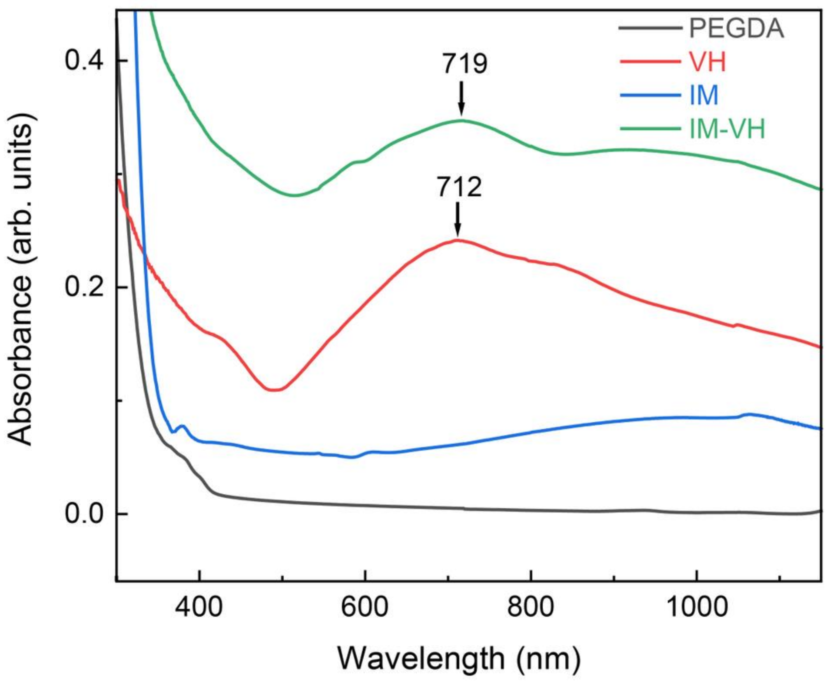

Supplementary Fig. 11. Optical absorption spectra for the IM, VH, IM-VH and PEGDA.

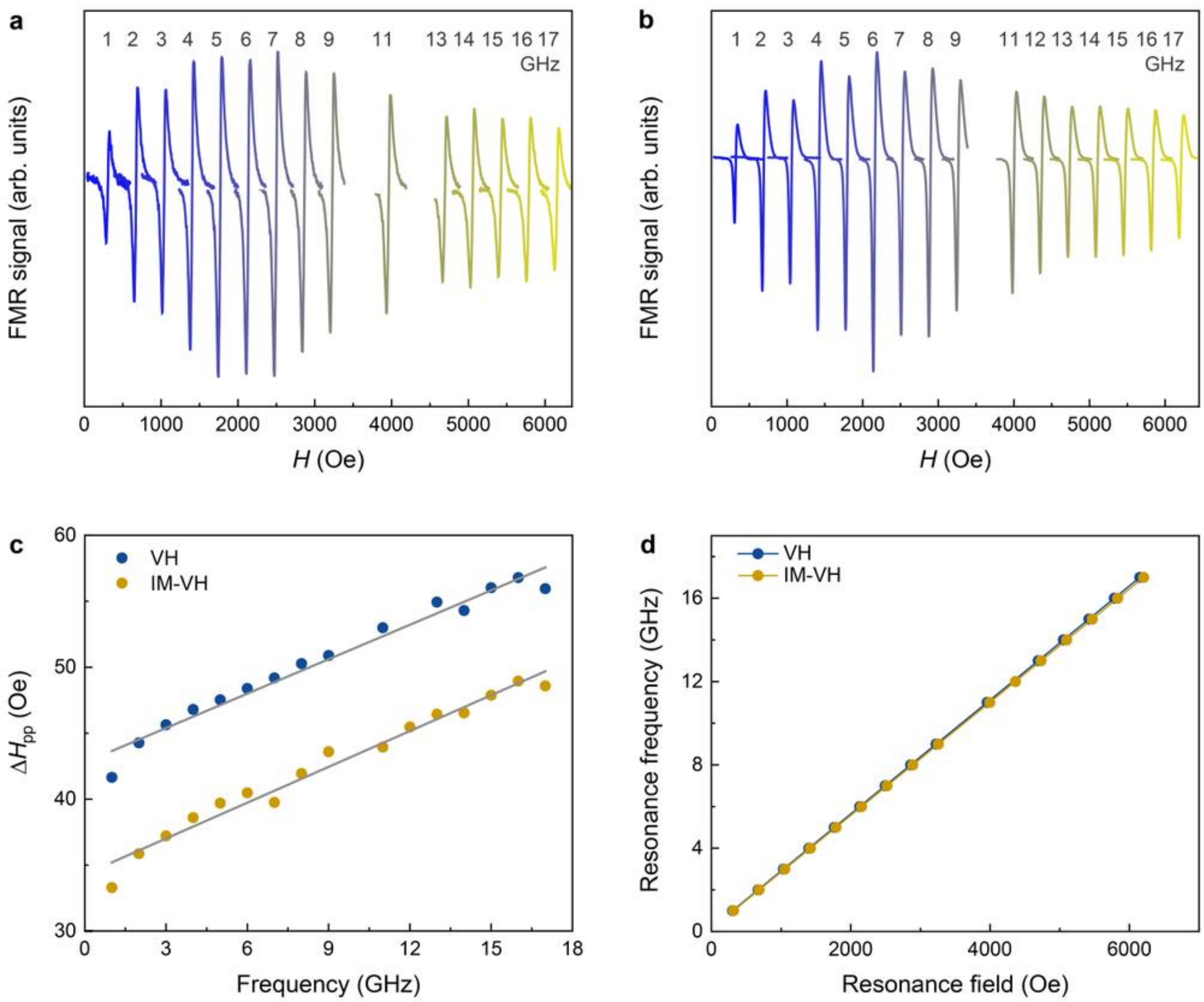

Supplementary Fig. 12. Room-temperature FMR spectrum for **a**, VH and **b**, IM-VH. **c**, Resonance linewidth (peak to peak, $\triangle H_{pp}$) as a function of frequency for VH and IM-VH. Solid lines are the fitting results using $\Delta H(\omega)=\Delta H(0)+\frac{2\alpha\omega}{\sqrt{3}\gamma}$ [21]. $\Delta H(\omega)$ and $\Delta H(0)$ is the frequency dependent and independent peak-to-peak linewidth. α is Gilbert damping constant. γ is the gyromagnetic ratio. ω is the resonance frequency. **d**, Resonance frequency as a function of resonance field.

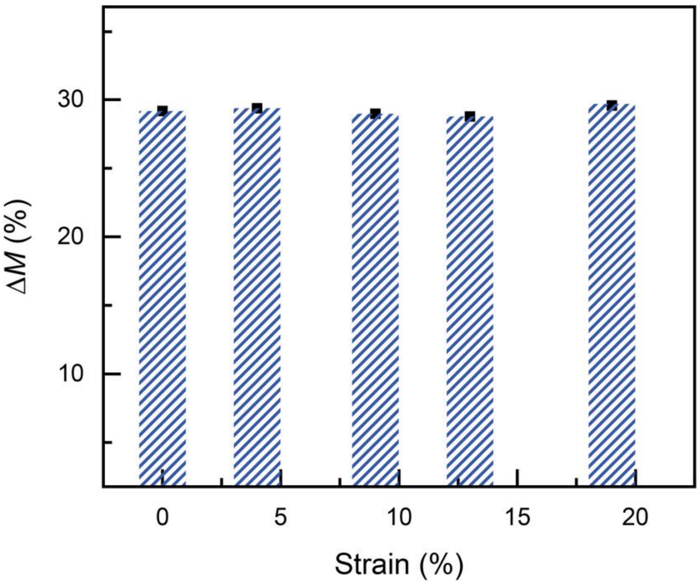

Supplementary Fig. 13. Magnetization change for IM-VH with different weight ratios at around low-temperature dielectric transition under 10Oe.

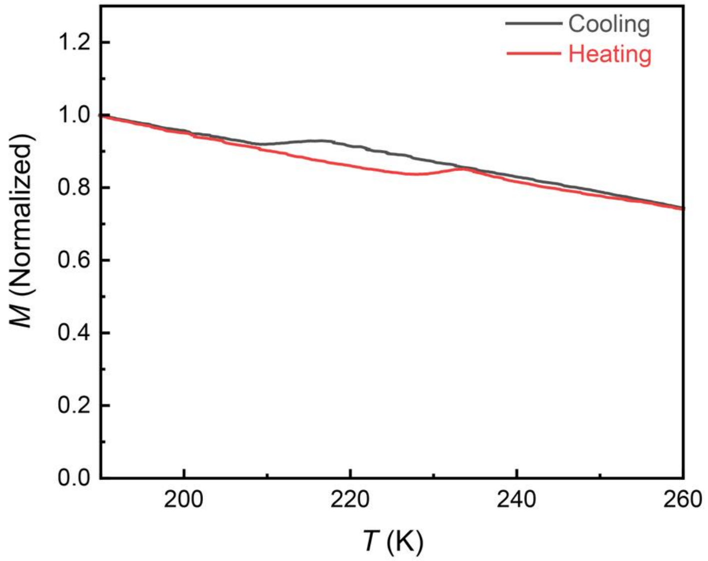

Supplementary Fig. 14. Temperature dependence of normalized magnetization for IM-VH pellet under 100 Oe.

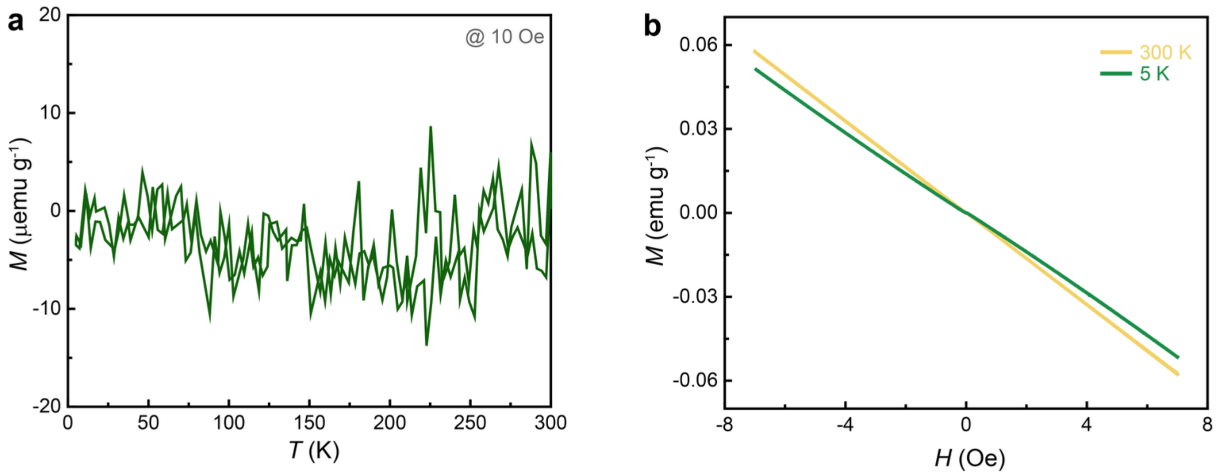

Supplementary Fig. 15. a, Temperature dependence of magnetization for IM. **b**, M-H loops for molecular ferroelectric IM.

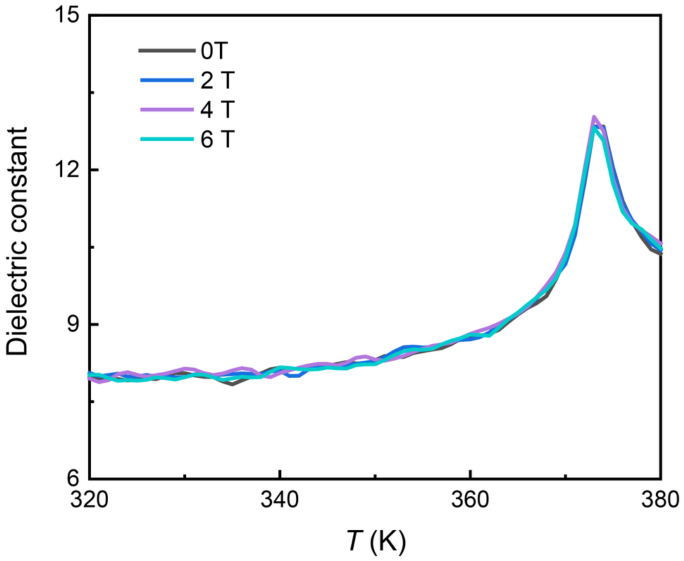

Supplementary Fig. 16. Temperature dependence of dielectric constant (1kHz) at different magnetic field for IM-VH.

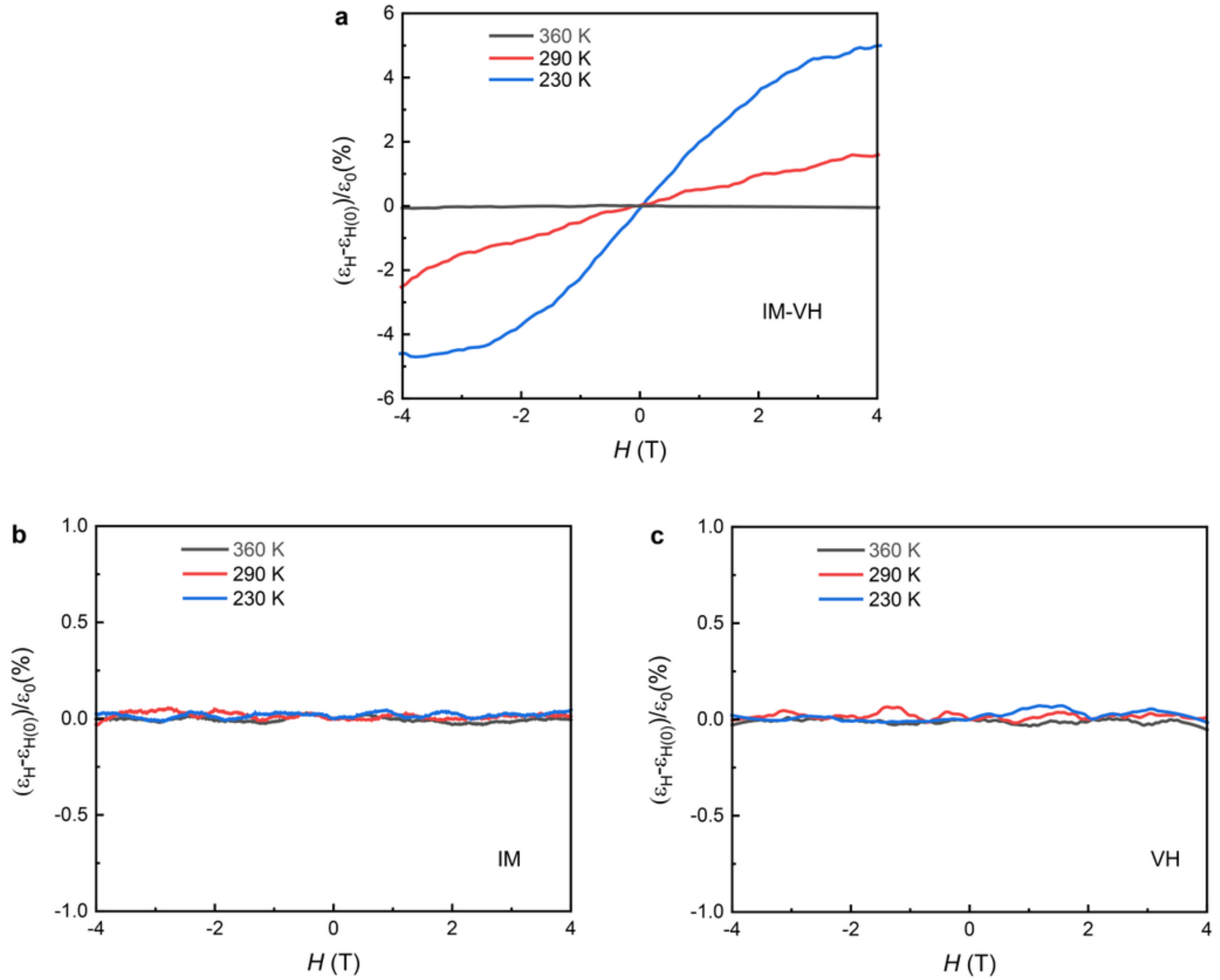

Supplementary Fig. 17. Magnetic-field dependence of permittivity (at 1 kHz) change for **a**, IM-VH, **b**, IM and **c**, VH.

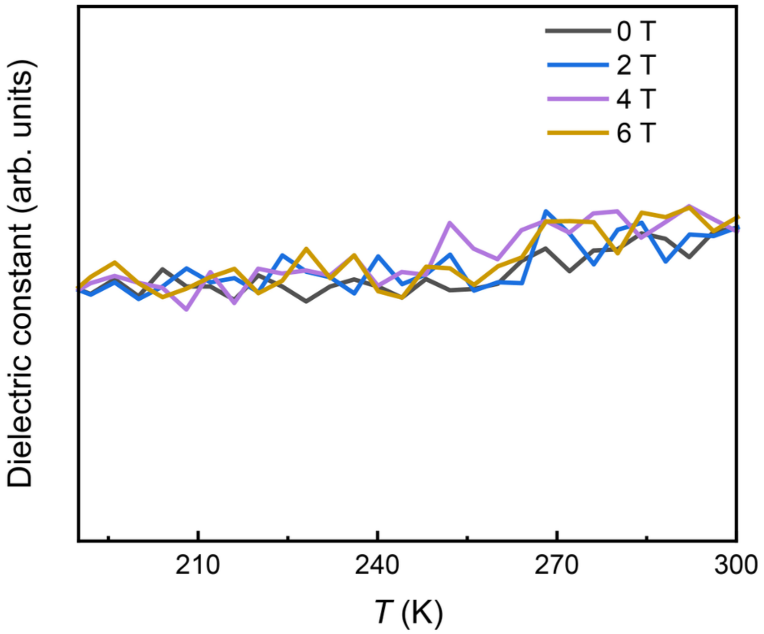

Supplementary Fig. 18. Temperature dependence of dielectric constant (1kHz) at different magnetic field for VH.

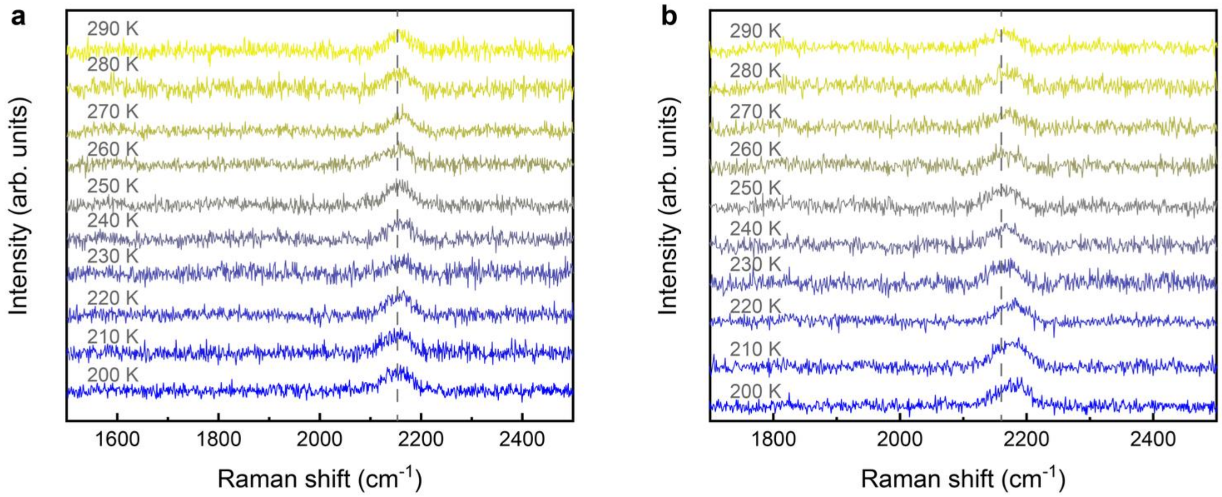

Supplementary Fig. 19. Temperature dependence of Raman spectra for **a**, VH and **b**, IM-VH.

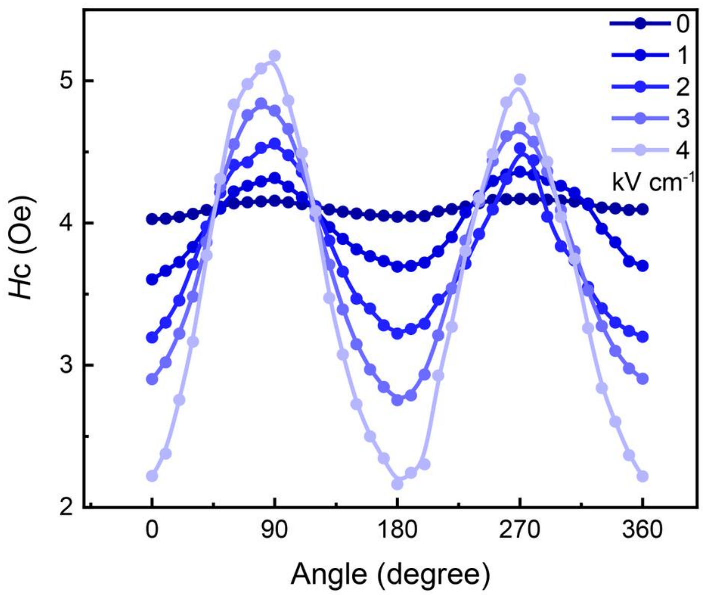

Supplementary Fig. 20. Room-temperature angle dependence of coercivity for printed IM-VH at different electric field. The horizontal axis shows the angle value between the applied magnetic field and magnetic field direction.

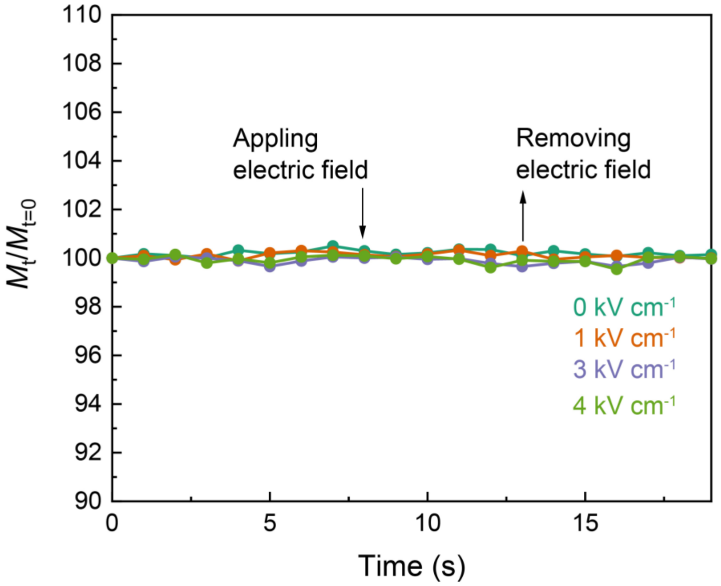

Supplementary Fig. 21. Room-temperature electric field control of magnetism for VH. The sample was first magnetized at 1T and then the magnetic field was removed. The electric field was applied along the magnetization direction and the remnant magnetization was monitoring in real time.

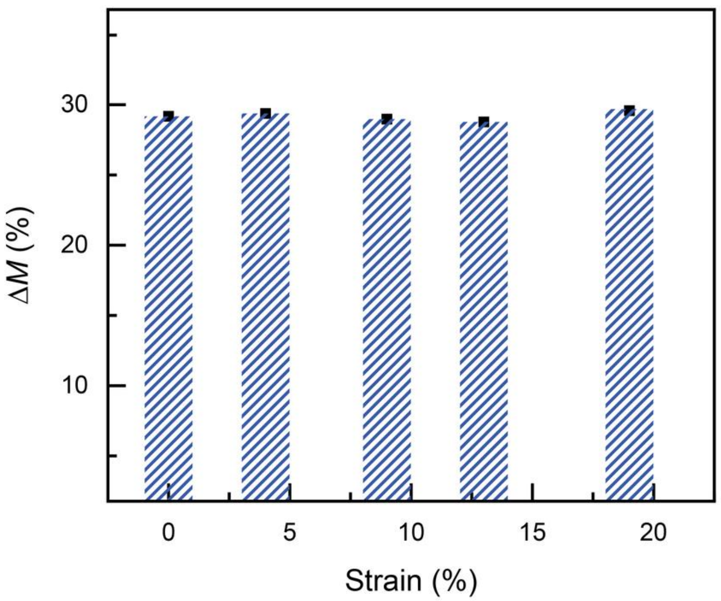

Supplementary Fig. 22. Electric field (4kV/cm) induced remnant magnetization change for IM-VH under different strain. The sample was compressed at different strain levels. The sample was first magnetized at 1T and then the magnetic field was removed. The electric field was applied along the magnetization direction and the remnant magnetization was monitoring in real time.

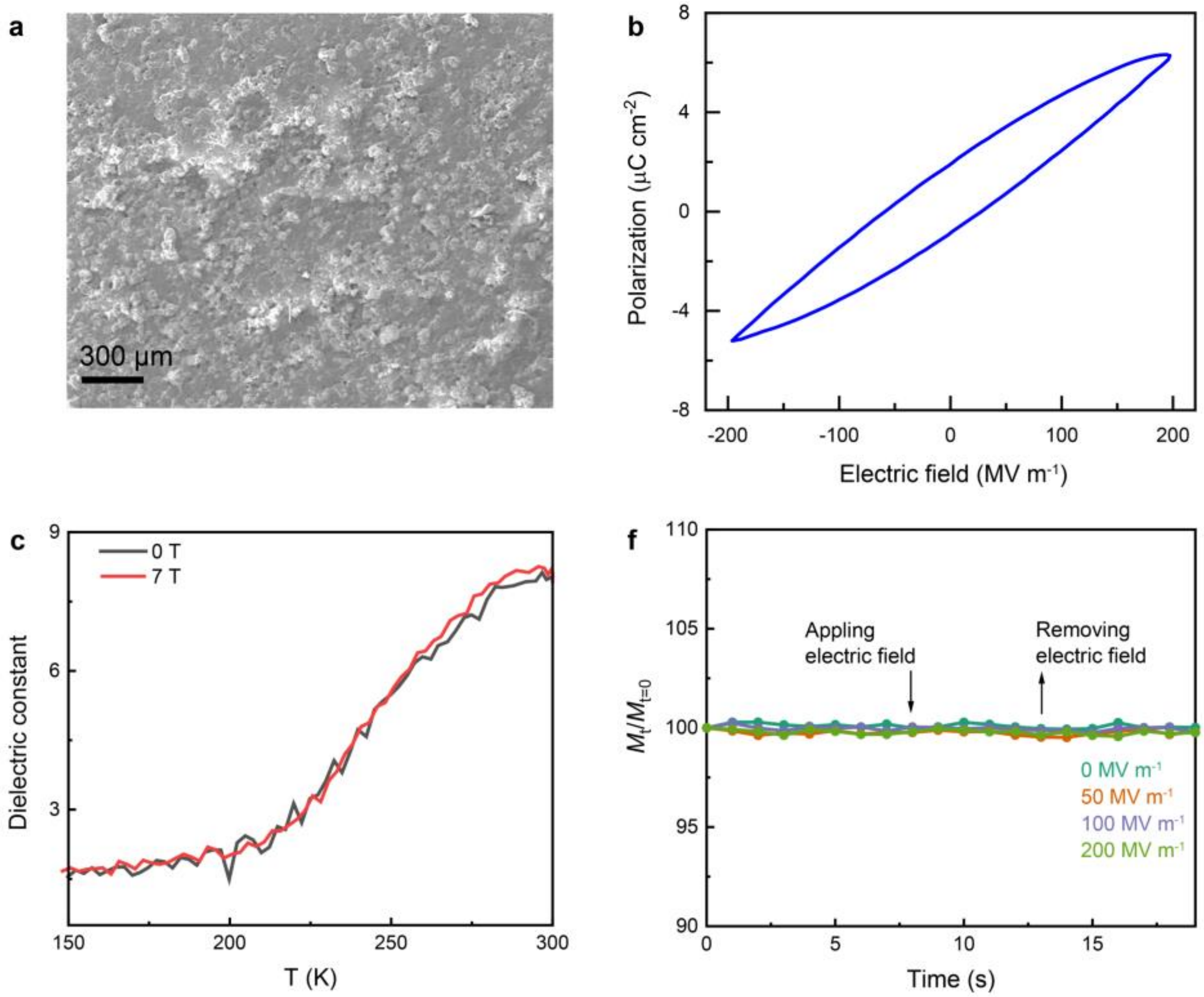

Supplementary Fig. 23. a, SEM image for PVDF-IM thin film composite. **b**, Polarization vs electric field for PVDF-VH thin film composite. **c**, Temperature dependence of dielectric constant (1kHz) at different magnetic fields for PVDF-VH thin film composite. **d**, Room-temperature electric field control of magnetism for PVDF-VH. The sample was first magnetized at 1T and then the magnetic field was removed. The electric field was applied along the magnetization direction and the remnant magnetization was monitoring in real time.

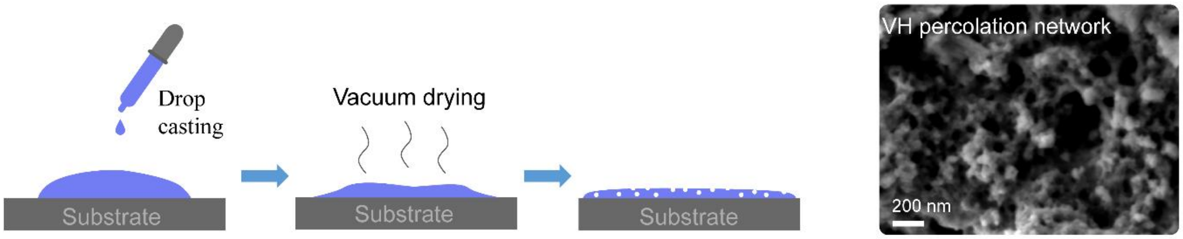

Supplementary Fig. 24. Thin-film vacuum drying method for creating nano-scale percolation network in the VH.

Supplementary Table S1. **T_c and coercive fields for selected high-temperature molecular magnets.**
TCNE: tetracyanoethylene; pyz: pyrazine; THF: tetrahydrofuran.

Compound	T_c (K)	Room-temperature H_c (Oe)	Reference
$Li_{0.7}[Cr(pyz)_2]Cl_{0.7}\cdot(THF)$	510	5300	22
$Li_{0.7}[Cr(pyz)_2]Cl_{0.7}\cdot 0.25(THF)$	515	7500	22
$V[TCNE]_x\cdot yCH_2Cl_2$ (x ~ 2; y ~ 0.5)	>350	60	23,24
$V[TCNE]_x$ (x ~ 2) thin films	400	～4.5	25
$V[Cr^{III}(CN)_6]_{0.86}\cdot 2.8H_2O$	315	≦25	26
$KV^{II}[Cr^{III}(CN)_6]\cdot 2H_2O$	376	～4	27
$V[Cr(CN)_6]_{0.87}\cdot 1.6H_2O$	360	～4.2	this work

Supplementary Table S2. **T_c, and piezoelectric coefficient (d_{33}) (pCN^{-1}) for selected high-temperature molecular ferroelectrics.**

	Molecular crystals	Piezoelectric coefficient (d_{33}) (pCN^{-1})	T_c (K)	Dissolvable in water?	Reference
1	Imidazolium Perchlorate （IM）	41	373	Y	28
2	Triglycine sulfate	35	322	Y	29
3	tetrathiafulvalene-p-chloranil (TTF-CA)	Not reported	81	N	30
4	trimethylbromomethylammonium tribromomanganese(II) (TMBM-$MnBr_3$)	112	415	Y	31
5	Guanidinium perchlorate	10	454	Y	32
6	$[Hdabco]ClO_4$; Hdabco = monoprotonated 1,4-diazabicyclo[2.2.2]octane	Not reported	377	Y	33

7	[Hdabco]BF_4;<br>Hdabco = monoprotonated 1,4-diazabicyclo[2.2.2]octane	Not reported	374	Y	34
8	[gua]ClO_4; gua = guanidinium	15	454	Y	35
9	[Et_4N]ClO_4; Et_4N = tetraethylammonium	Not reported	378	Y	36
10	[qui]IO_4; qui = quinuclidinium	Not reported	322	Y	37
11	[hqu]Cl;<br>hqu = (R)-(−)-3-hydroxlyquinuclidinium	6	340	Y	38
12	[apd]$RbBr_3$;<br>apd = 3-ammoniopyrrolidinium	Not reported	440	Y	39
13	[MeHdabco]RbI_3;<br>MeHdabco = protonated N-methyl-1,4-diazoniabicyclo[2.2.2]octane	Not reported	430	Y	40
14	[tmno]$_2$[KFe$(CN)_6$] ;<br>tmno = protonated trimethylamine N-oxide	Not reported	402	Y	41
15	[tmcm]$MnCl_3$;<br>tmcm = trimethylchloromethylammonium	185	406	Y	42
16	Diisopropylammonium bromide	/	426	Y	43
17	1-Azabicyclo[2.2.1]heptanium perrhenate	90	322	Y	44
18	Poly(vinylidene fluoride), (PVDF)	28	363	N	45
19	PVDF–TrFE	38	373	N	45
20	Parylene-C	2	/	N	46
21	diisopropylammonium bromide	18	425	Y	47
22	tetramethylammonium tetrachloroferrate(III)	80	344	Y	48
23	tetramethylammonium bromotrichloroferrate-(III)	110	346	Y	48
24	tetramethylammonium tetrachlorogallate(III)	80	383	Y	49
25	Croconic acid	15.1	420	Y	50
26	phenylmalonaldehyde (PhMDA)	10.4	363	N	50,51
27	5,6-dichloro-2-methyl-1H-benzimidazole (DC-MBI)	12.2	399	N	50,52
28	2-methyl-1Hbenzimidazole (MBI)	7.5	430	N	50,52
29	Triglycine sulfate (TGS)	29.1	323	Y	53
30	3-hydroxy-1H-phenalen-1-one (HPLN)	7.3	420	Y	50,51

Supplementary Table S3. **3D printing process parameters**

Light Intensity	68 μW cm^{-2}
Separation Speed	0.05 mm s^{-1}
Layer thickness	50 μm

Supplementary Table S4. **FMR parameters**

Samples	Gamma (GHz T^{-1})	Gilbert damping constant, (10^{-3})
VH	171.9	2.06
IM-VH	170.7	2.13

Supplementary References

1 Bicerano, J. *Prediction of polymer properties*. (cRc Press, 2002).

2 Van Krevelen, D. W. & Te Nijenhuis, K. *Properties of polymers: their correlation with chemical structure; their numerical estimation and prediction from additive group contributions*. (Elsevier, 2009).

3 Mullis, A. S. *et al.* Data Analytics Approach for Rational Design of Nanomedicines with Programmable Drug Release. *Mol. Pharm.* **16**, 1917-1928 (2019).

4 Ulery, B. D. *et al.* Rational design of pathogen-mimicking amphiphilic materials as nanoadjuvants. *Sci. Rep.* **1**, 1-9 (2011).

5 Li, X., Petersen, L., Broderick, S., Narasimhan, B. & Rajan, K. Identifying factors controlling protein release from combinatorial biomaterial libraries via hybrid data mining methods. *ACS combinatorial science* **13**, 50-58 (2011).

6 Tenenbaum, J. B., De Silva, V. & Langford, J. C. A global geometric framework for nonlinear dimensionality reduction. *Science* **290**, 2319-2323 (2000).

7 Shen, X., Zhang, T., Broderick, S. & Rajan, K. Correlative analysis of metal organic framework structures through manifold learning of Hirshfeld surfaces. *Molecular Systems Design & Engineering* **3**, 826-838 (2018).

8 Srinivasan, S. *et al.* Mapping Chemical Selection Pathways for Designing Multicomponent Alloys: an informatics framework for materials design. *Sci. Rep.* **5**, 1-8 (2015).

9 Wagaman, A. & Levina, E. Discovering sparse covariance structures with the isomap. *Journal of Computational and Graphical Statistics* **18**, 551-572 (2009).

10 Cukierski, W. J. & Foran, D. J. in *Data Mining Workshops, 2008. ICDMW'08. IEEE International Conference on.* 949-958 (IEEE).

11 Balasubramanian, M., Schwartz, E. L., Tenenbaum, J. B., de Silva, V. & Langford, J. C. The isomap algorithm and topological stability. *Science* **295**, 7-7 (2002).

12 Wold, S., Sjostrom, M. & Eriksson, L. PLS-regression: a basic tool of chemometrics. *Chemometrics and Intelligent Laboratory Systems* **58**, 109-130 (2001).

13 Nguyen, D. V. & Rocke, D. M. Vol. 18 39-50 (2002).

14 Rosipal, R. & Kramer, N. in *Subspace, Latent Structure and Feature Selection Techniques* (eds C. Saunders, M. Grobelnik, S. Gunn, & J. Shawe-Taylor) 34-51 (Springer, 2006).

15 Geladi, P. & Kowalski, B. R. Partial least-squares regression: a tutorial. *Analytica Chimica Acta* **185**, 1-17 (1986).

16 de Jong, S. SIMPLS: An alternative approach to partial least squares regression. *Chemometrics and Intelligent Laboratory Systems* **18**, 251-263 (1993).

17 Phatak, A. & Jong, S. D. The geometry of partial least squares. **11**, 311-338 (1997).

18 Ferlay, S., Mallah, T., Ouahes, R., Veillet, P. & Verdaguer, M. A room-temperature organometallic magnet based on Prussian blue. *Nature* **378**, 701 (1995).

19 Hatlevik, Ø., Buschmann, W. E., Zhang, J., Manson, J. L. & Miller, J. S. Enhancement of the magnetic ordering temperature and air stability of a mixed valent vanadium hexacyanochromate (III) magnet to 99 ℃ (372 K). *Adv. Mater.* **11**, 914-918 (1999).

20 Garde, R., Villain, F. & Verdaguer, M. Molecule-based room-temperature magnets: catalytic role of V (III) in the synthesis of vanadium− chromium Prussian blue analogues. *J. Am. Chem. Soc.* **124**, 10531-10538 (2002).

21 Oates, C. *et al.* High field ferromagnetic resonance measurements of the anisotropy field of longitudinal recording thin-film media. *J. Appl. Phys.* **91**, 1417-1422 (2002).
22 Perlepe, P. *et al.* Metal-organic magnets with large coercivity and ordering temperatures up to 242 ℃. *Science* **370**, 587-592 (2020).
23 Sieklucka, B. & Pinkowicz, D. *Molecular magnetic materials: concepts and applications*. (John Wiley & Sons, 2017).
24 Manriquez, J. M., Yee, G. T., McLean, R. S., Epstein, A. J. & Miller, J. S. A room-temperature molecular/organic-based magnet. *Science* **252**, 1415-1417 (1991).
25 Pokhodnya, K. I., Epstein, A. J. & Miller, J. S. Thin-Film V [TCNE] x Magnets. *Adv. Mater.* **12**, 410-413 (2000).
26 Ferlay, S., Mallah, T., Ouahes, R., Veillet, P. & Verdaguer, M. A room-temperature organometallic magnet based on Prussian blue. *Nature* **378**, 701-703 (1995).
27 Holmes, S. M. & Girolami, G. S. Sol− Gel Synthesis of KVII [CrIII (CN) 6]⊙ 2H2O: A Crystalline Molecule-Based Magnet with a Magnetic Ordering Temperature above 100 °C. *J. Am. Chem. Soc.* **121**, 5593-5594 (1999).
28 Zhang, Y. *et al.* A molecular ferroelectric thin film of imidazolium perchlorate that shows superior electromechanical coupling. *Angew. Chem. Int. Ed.* **53**, 5064-5068 (2014).
29 Luo, E., Xie, Z., Xu, J., Wilson, I. & Zhao, L. In situ observation of the ferroelectric-paraelectric phase transition in a triglycine sulfate single crystal by variable-temperature electrostatic force microscopy. *Phys. Rev. B* **61**, 203 (2000).
30 Kobayashi, K. *et al.* Electronic ferroelectricity in a molecular crystal with large polarization directing antiparallel to ionic displacement. *Phys. Rev. Lett.* **108**, 237601 (2012).
31 Liao, W. Q., Tang, Y. Y., Li, P. F., You, Y. M. & Xiong, R. G. Large Piezoelectric Effect in a Lead-Free Molecular Ferroelectric Thin Film. *J. Am. Chem. Soc.* **139**, 18071-18077 (2017).
32 Pan, Q. *et al.* A Molecular Polycrystalline Ferroelectric with Record-High Phase Transition Temperature. *Adv. Mater.* **29** (2017).
33 Tang, Y.-Y. *et al.* Ultrafast polarization switching in a biaxial molecular ferroelectric thin film:[Hdabco] ClO4. *J. Am. Chem. Soc.* **138**, 15784-15789 (2016).
34 Shi, P.-P., Tang, Y.-Y., Li, P.-F., Ye, H.-Y. & Xiong, R.-G. De novo discovery of [Hdabco] BF4 molecular ferroelectric thin film for nonvolatile low-voltage memories. *J. Am. Chem. Soc.* **139**, 1319-1324 (2017).
35 Pan, Q. *et al.* A Molecular Polycrystalline Ferroelectric with Record-High Phase Transition Temperature. *Adv. Mater.* **29**, 1700831 (2017).
36 Ye, H.-Y. *et al.* Molecular ferroelectric with most equivalent polarization directions induced by the plastic phase transition. *J. Am. Chem. Soc.* **138**, 13175-13178 (2016).
37 You, Y.-M. *et al.* Quinuclidinium salt ferroelectric thin-film with duodecuple-rotational polarization-directions. *Nat. Commun.* **8**, 1-7 (2017).
38 Li, P.-F. *et al.* Anomalously rotary polarization discovered in homochiral organic ferroelectrics. *Nat. Commun.* **7**, 1-9 (2016).
39 Pan, Q. *et al.* A three-dimensional molecular perovskite ferroelectric:(3-ammoniopyrrolidinium) RbBr3. *J. Am. Chem. Soc.* **139**, 3954-3957 (2017).
40 Zhang, W.-Y. *et al.* Precise Molecular Design of High-T c 3D Organic–Inorganic Perovskite Ferroelectric:[MeHdabco] RbI3 (MeHdabco= N-Methyl-1, 4-diazoniabicyclo [2.2. 2] octane). *J. Am. Chem. Soc.* **139**, 10897-10902 (2017).

41 Xu, W.-J. *et al.* A molecular perovskite with switchable coordination bonds for high-temperature multiaxial ferroelectrics. *J. Am. Chem. Soc.* **139**, 6369-6375 (2017).

42 You, Y.-M. *et al.* An organic-inorganic perovskite ferroelectric with large piezoelectric response. *Science* **357**, 306-309 (2017).

43 Fu, D.-W. *et al.* Diisopropylammonium bromide is a high-temperature molecular ferroelectric crystal. *Science* **339**, 425-428 (2013).

44 Harada, J. *et al.* Plastic/ferroelectric crystals with easily switchable polarization: low-voltage operation, unprecedentedly high pyroelectric performance, and large piezoelectric effect in polycrystalline forms. *J. Am. Chem. Soc.* **141**, 9349-9357 (2019).

45 Ramadan, K. S., Sameoto, D. & Evoy, S. A review of piezoelectric polymers as functional materials for electromechanical transducers. *Smart Mater. Struct.* **23**, 033001 (2014).

46 Kim, J. Y.-H., Cheng, A. & Tai, Y.-C. in *2011 IEEE 24th International Conference on Micro Electro Mechanical Systems.* 473-476 (IEEE).

47 Yadav, H., Sinha, N., Goel, S., Hussain, A. & Kumar, B. Growth and structural and physical properties of diisopropylammonium bromide molecular single crystals. *J. Appl. Crystallogr.* **49**, 2053-2062 (2016).

48 Harada, J. *et al.* Ferroelectricity and piezoelectricity in free-standing polycrystalline films of plastic crystals. *J. Am. Chem. Soc.* **140**, 346-354 (2018).

49 Li, D. *et al.* Construction of Magnetoelectric Composites with a Large Room-Temperature Magnetoelectric Response through Molecular–Ionic Ferroelectrics. *Adv. Mater.* **30**, 1803716 (2018).

50 Horiuchi, S., Tsutsumi, J. y., Kobayashi, K., Kumai, R. & Ishibashi, S. Piezoelectricity of strongly polarized ferroelectrics in prototropic organic crystals. *J. Mater. Chem. C* **6**, 4714-4719 (2018).

51 Horiuchi, S., Kumai, R. & Tokura, Y. Hydrogen-bonding molecular chains for high-temperature ferroelectricity. *Adv. Mater.* **23**, 2098-2103 (2011).

52 Horiuchi, S. *et al.* Above-room-temperature ferroelectricity and antiferroelectricity in benzimidazoles. *Nat. Commun.* **3**, 1-6 (2012).

53 Pandian, M. S., Ramasamy, P. & Kumar, B. A comparative study of ferroelectric triglycine sulfate (TGS) crystals grown by conventional slow evaporation and unidirectional method. *Mater. Res. Bull.* **47**, 1587-1597 (2012).
