# Supplementary material for: Proton switching molecular magnetoelectricity
Source: Nat Commun. 2021 Jul 29;12:4602. doi: 10.1038/s41467-021-24941-9 (PMC8322162; doi:10.1038/s41467-021-24941-9)
Supplement: Supplementary file 2 — Description of Additional Supplementary Files [file 41467_2021_24941_MOESM2_ESM.pdf]

Description of Additional Supplementary Files

File Name: Supplementary Movie 1
Description: The ultraviolet (UV) light has a wavelength of 385 nm and the control of image projection was achieved through a dynamic micro-mirror device. The precursor was exposed to the UV light, and the PEGDA was then cross-linked to form the scaffold network with the encapsulated ions and PBA particles. As shown in the video, a complex geometric structure (Lattice structure, 25×25×25 mm^3) can be printed.
